# Comprehensive Analysis of m6A RNA Methylation Regulators and the Immune Microenvironment to Aid Immunotherapy in Pancreatic Cancer

**DOI:** 10.3389/fimmu.2021.769425

**Published:** 2021-11-05

**Authors:** Yongdong Guo, Ronglin Wang, Junqiang Li, Yang Song, Jie Min, Ting Zhao, Lei Hua, Jingjie Shi, Chao Zhang, Peixiang Ma, Cheng Yang, Liaoliao Zhu, Dongxue Gan, Shanshan Li, Xiaonan Liu, Haichuan Su

**Affiliations:** ^1^ Department of Oncology, Tangdu Hospital, Air Force Medical University, Xi'an, China; ^2^ Department of Ambulatory Surgery Center, Xijing Hospital, Air Force Medical University, Xi'an, China

**Keywords:** pancreatic cancer, m6A regulators, immune microenvironment, immunotherapy, prognosis

## Abstract

Pancreatic cancer (PAAD) is one of the most malignant cancers and immune microenvironment has been proved to be involved in pathogenesis of PAAD. m6A modification, related to the expression of m6A regulators, participates in the development of multiple cancers. However, the correlation between m6A regulators and immune microenvironment was largely unknown in PAAD. And because of the small sample size of pancreatic cancer in the TCGA database, it is not enough to draw a convincing conclusion. In the present study, we downloaded seven pancreatic cancer datasets with survival data and removed batch effects among these datasets to be used as the PAAD cohort to analyze the immune landscape of PAAD and the expression pattern of m6A regulators and divided the integrated dataset into cluster 1 and cluster 2 by consensus clustering for m6A regulators. Lower m6A regulators were found to be related to higher immune cell infiltration and a better survival. Moreover, we identified six m6A regulators and constructed the prognostic signature of m6A regulators. Patients with low-risk score had a higher response to immune checkpoint inhibitor and a longer overall survival. To figure out the underlying mechanism, we analyzed the cancer immunity cycle, most altered genes, gene set enrichment analysis (GSEA) and gene set variation analysis (GSVA) in risk subtypes. In summary, the present study proved m6A regulators modulated the PAAD immune microenvironment. And risk scores served as predictive indicator for immunotherapy and played a prognostic role for PAAD patients. Our study provided novel therapeutic targets to improve immunotherapy efficacy.

## Introduction

Pancreatic adenocarcinoma (PAAD) is one of the most malignant cancers in the world and has a poor prognosis (1). Surgical resection is the primary method to treat PAAD. However, 90% of patients have already developed metastasis when they are diagnosed, thus preventing the opportunity for surgical resection. Chemotherapy, molecular targeted therapy, and radiotherapy are the canonical treatments for PAAD patients. Despite the accumulating developments in early diagnosis and treatment, the 5-year survival rate of PAAD patients is only approximately 4% (2). Great efforts are urgently needed to explore novel therapeutic strategies for PAAD.

Accumulating evidence has shown that the tumor immune microenvironment plays a critical role in the pathogenesis of PAAD (3). PAAD patients presented increased immunosuppressive cells, impaired natural killer (NK) cell activity, inactivated cytotoxic T lymphocytes and other dysregulated immune cells. PAAD was revealed as a “cold tumor” with limited infiltrating immune cells (4). Immunotherapy has been widely acknowledged as a promising treatment in many cancer types due to its safety and efficacy. However, only a fraction of PAAD patients are sensitive to immunotherapy (5, 6). There is an urgent need to determine the underlying molecular mechanism for immunotherapy resistance and to confirm appropriate biomarkers to speculate about the immunotherapy benefit for PAAD patients.

N6-Methyladenosine (m6A) refers to the methylation modification at the sixth N atom of adenine, which has been validated as the most common posttranscriptional modification on mRNA (7). By modulating the transcription, translation process or stability of target mRNAs, m6A modification regulates multiple cellular activities, including the immune response and the tumor immune microenvironment. m6A modification has been proven to promote the development of PAAD and to mediate chemotherapy resistance (8, 9). m6A modification is related to the expression of m6A regulators, including methyltransferases (“writers”), demethylases (“erasers”), and binding proteins (“readers”). Previous studies have indicated that m6A regulators play diagnostic and prognostic roles in many cancer types (10, 11). Wang et al. (12) found that the m6A levels were elevated in approximately 70% of the PAAD samples and METTL14 overexpression markedly promoted PAAD cell proliferation and migration both *in vivo* and *in vitro*. Guo et al. (13) uncovered that ALKBH5 serve as a suppressor by regulating the posttranscriptional activation of PER1 through m6A abolishment. What’s more, Meng et al. (14) identified differentially expressed genes between PAAD groups with/without genetic alteration of m6A regulators, and then generated 16-mRNA signature score used to be a promising prognostic indicator. Some predictive models based on these regulators help to infer the immunotherapy benefits for patients. However, it remains to be fully explored whether m6A regulators are related to the immune microenvironment. What are the effects of m6A regulators on immune cell infiltration? What are the underlying molecular pathways? Can m6A regulators be utilized as biomarkers for PAAD immunotherapy efficacy? These questions all remain to be answered.

In the present study, we analyzed m6A regulator expression across cancers and 930 PAAD patients. Genetic and transcriptional alterations of m6A regulators in PAAD were examined. We classified PAAD patients into different subtypes and analyzed their relationship with the tumor microenvironment. Moreover, we constructed risk scores to characterize the various immune landscapes. This model could assist in precisely predicting PAAD patient prognosis and predicting immunotherapy benefits.

## Results

### Pan-Cancer Expression Alterations of m6A Regulators

**Figure 1:**
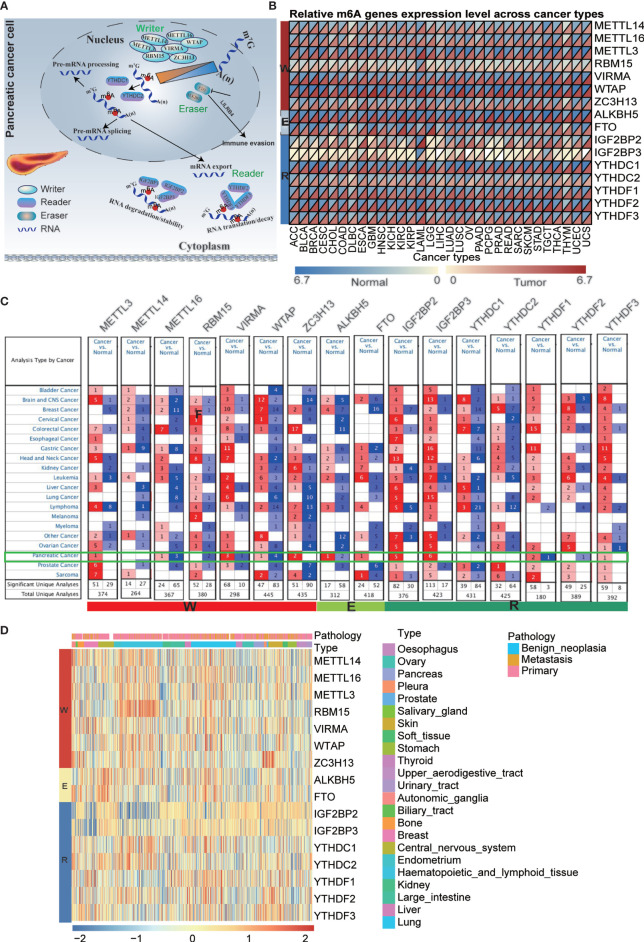
Pan-cancer expression and alterations of m6A regulators. **(A)** Diagram of m6A regulators modification and its biological function. **(B)** The gene expression of m6A regulators in 31 cancer types based on TCGA + GTEx databases. **(C)** The gene expression of m6A regulators in 21 cancer types based on oncomine database, which integrates RNA and DNA-seq data from GEO, TCGA and published literatures. **(D)** The gene expression of m6A regulators in 23 cancer cell lines based on CCLE database.

It has been reported that m6A regulators are composed of three types of regulatory proteins: methyltransferases (“writers”), demethylases (“erasers”) and binding proteins (“readers”). The writers mainly mediate m6A RNA methylation, while the erasers play the opposite role. The “readers” specifically bind to the m6A methylation sites to perform the function. These proteins cooperate to modulate target gene expression and function and are thus involved in the pathogenesis, development of tumors, and immune evasion (**Figure 1A**). To explore the underlying mechanism, we investigated the 16 m6A regulator expression alterations in 31 cancer types based on the available datasets from The Cancer Genome Atlas (TCGA) and The Genotype-Tissue Expression (GTEx) database (**Figures 1B** and **Tables S2**, **S3**), 21 cancer types based on the Oncomine database and 23 cancer cell lines based on the CCLE database (**Figures 1C, D** and **Table S4**). Previous studies have mainly examined m6A regulator expression in TCGA datasets. In the present study, we further explored target gene expression in the Oncomine database, which contains not only the TCGA dataset but also datasets from previously published literature. The results showed that m6A regulator expression patterns are different between cancer tissues and normal tissues.

### The Landscape of Expression and Genetic and Transcriptional Alterations of m6a Regulators in Pancreatic Cancer

**Figure 2:**
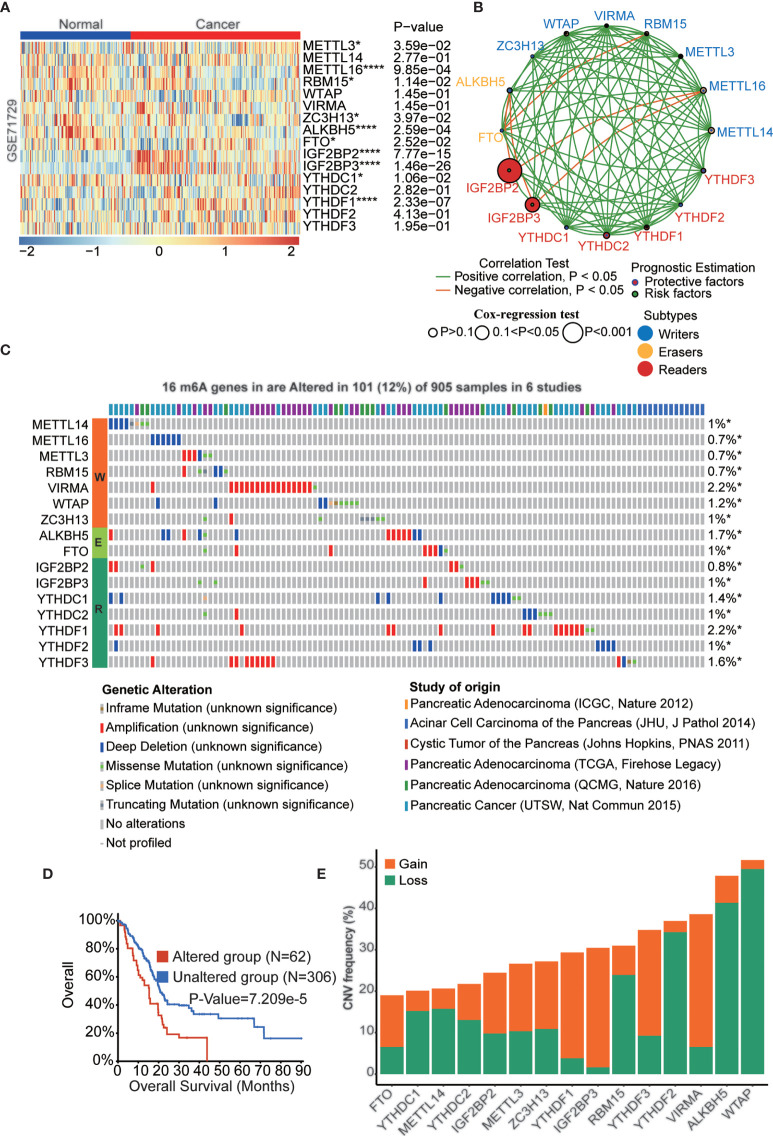
The landscape of genetic and transcriptional alterations of m6A regulators in pancreatic cancer. **(A)** The difference of mRNA expression levels of 16 m6A regulators between normal and PAAD samples. **(B)** The interaction of expression of 16 m6A regulators in PAAD. The m6A regulators in three RNA modification types were depicted by circles in different colors. The lines connecting m6A regulators represented their positive/negative correlation with each other. The size of each circle represented the prognosis effect of each regulator and scaled by P-value. **(C)** 101 of the 905 PAAD patients in 6 studies experienced genetic alterations of 16 m6A regulators. **(D)** Kaplan-Meier plots comparing OS in cases with and without 16 m6A regulators alterations in patients with PAAD. **(E)** the CNV alteration frequency of 16 m6A regulators in PAAD. *p < 0.05; ****p < 0.0001.

Pancreatic cancer is one of the most malignant cancers in the world with a poor prognosis. Previous studies have indicated that m6A regulators play critical roles in pancreatic cancer development, while the function and mechanism of these proteins remain to be fully elucidated. We downloaded 5 pancreatic cancer datasets with survival data (GSE28735, GSE57495, GSE62452, MTAB-6134, and TCGA-PAAD) and removed batch effects among these datasets to be used as the training cohort (**Figures S1A, B**). Similarly, datasets ICGC-AU and ICGC-CA were integrated as the validation cohort (**Figures S1C, D**). m6A regulator expression was analyzed in pancreatic cancer and normal samples in the GSE71729 dataset, which indicated that most of these genes were dysregulated in PAAD tissues. (**Figure 2A**). To further confirm the expression patterns in PAAD, we assessed m6A regulator mRNA expression among normal, tumor and metastatic tissues based on the TNMplot database and protein expression based on the Human Protein Atlas (HPA) database (**Figures S2**, **S3**). Interestingly, the results indicated that most of these genes have lower expression in metastatic tissues than primary tissues in PAAD patients. The expression of m6A regulators was also analyzed in PAAD cell lines (**Figures S4A, B**). m6A regulators cooperate to regulate the m6A modification level of target genes and then affect cellular homeostasis. As shown in **Figure 2B**, a complex network existed among m6A regulators in PAAD. For example, IGF2BP2 and IGF2BP3 had a negative correlation with METTL16. Furthermore, we examined the clinical significance of m6A regulators in PAAD, and the results showed that most of these genes were significantly correlated with OS of PAAD patients (**Figure S4C**). Genetic alteration is a critical factor influencing gene expression and function. We found that 101 of the 905 PAAD patients in 6 studies experienced genetic alterations of 16 m6A regulators, and amplification was the most prevalent genetic alteration (**Figure 2C**). The OS (p<0.001) of the PAAD patients without 16 m6A regulator alterations was longer than that of patients with alterations (**Figure 2D**). Moreover, the DFS (p<0.001), DSS (p<0.05), and PFS (p<0.05) of the patients without 16 m6A regulator alterations were longer than those in PAAD patients with alterations (**Figures S4D-F**). Similarly, the CNV frequency of these m6A regulators was also tested in PAAD patients (**Figure 2E**).

### Identified Consensus Clustering Subtype and the Landscape of Immune Cell Infiltration in the TME of PAAD

**Figure 3:**
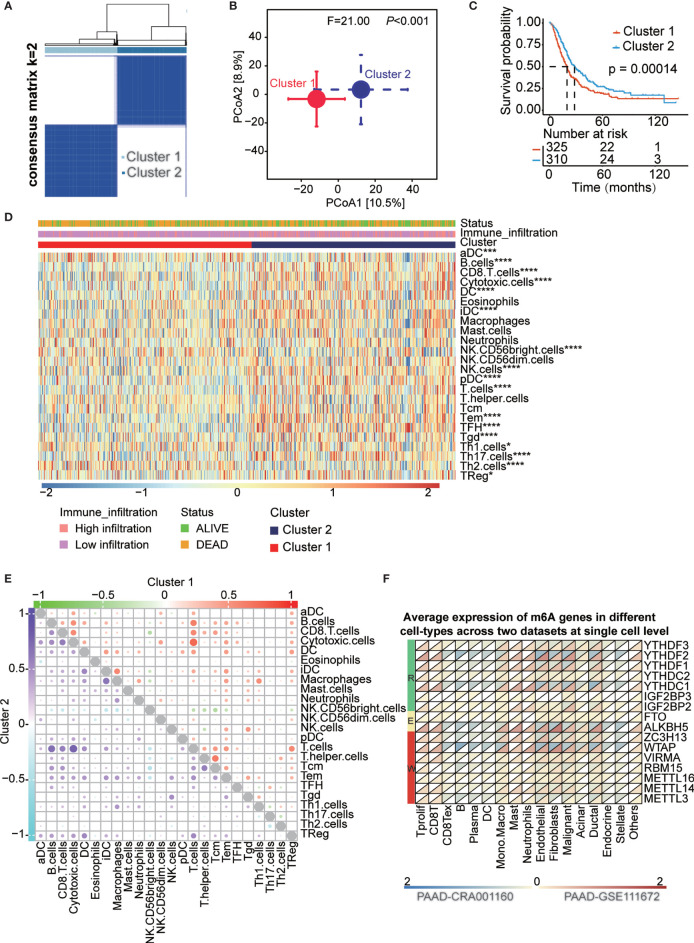
Survival in cluster1/2 subtypes and the landscape of immune infiltration in the TME of PAAD. **(A)** Consensus clustering matrix for k=2. **(B)** Principal component analysis confirmed the two clusters, Cluster 1 (red), Cluster 2 (blue). **(C)** Kaplan-Meier curves of overall survival (OS) for patients with PAAD in cluster1/2 subtypes. **(D)** The heatmap showed the comparison of fraction of tumor-infiltrating immune cells in two clusters. **(E)** The correlation of the infiltrating levels of each immune cell in cluster 1 and cluster 2 subtypes, respectively. Bubble size and color represents correlation coefficient r, when p-value > 0.05 without bubbles. **(F)** Average expression of 16 m6A genes in different cell-types at single cell level across two datasets. *p < 0.05; ***p < 0.001; ****p < 0.0001.

k = 2 was identified with optimal clustering stability from k = 2 to 9 based on the similarity displayed by the expression levels of 16 m6A regulators and the proportion of ambiguous clustering measures (**Figures 3A** and **S5A-C**). A total of 635 PAAD patients were divided into cluster 1 and cluster 2 based on the m6A regulator expression level (**Table S5**) and principal coordinates analysis (PCoA) method showed clear distinction in two cluster subgroups (p<0.001, **Figure 3B**). The OS (p<0.001) of patients in cluster 2 was longer than that of patients in cluster 1 (**Figure 3C**). The expression of most of m6A regulators was dysregulated among cluster subtypes (**Figure S5D**). For the heterogeneity of individuals, only a small proportion of patients will benefit from immunotherapy, while others are not sensitive to immunotherapy. Therefore, we analyzed a series of immune-related indicators to identify which group of PAAD patients are more suitable for immunotherapy. Immune cell infiltration has been proven to be a factor related to the therapy responsiveness and prognosis of PAAD patients. m6A regulators have been reported to participate in PAAD progression by regulating immune cell infiltration. In the present study, immune cell infiltration was estimated by single-sample gene set enrichment analysis (ssGSEA) and the deconvolution algorithm, and all patients were divided into high- and low-infiltration subtypes (**Figure S6A** and **Table S6**). We found that the OS (p<0.05) of the high infiltration subtype was longer than that of the low infiltration subtype (**Figure S6B**). We also explored the fraction of tumor-infiltrating immune cells in the two clusters. The patients in cluster 2 had higher immune cell infiltration than those in cluster 1 (**Figure 3D**). A complex regulatory network exists among immune cells. We analyzed the correlation of infiltrating immune cells in two cluster subtypes and two infiltration subtypes (**Figures 3E** and **Figures S6D**, **S5E**). The results showed that most immune cells are positively correlated with each other in two clusters. Additionally, we assessed the clinical significance of these tumor-infiltrating immune cells in PAAD and most of these cells significantly correlated with OS of PAAD patients (**Figure S6C**). To uncover the regulatory relationship between m6A regulators and infiltrating immune cells, we analyzed the correlation of infiltrating immune cells and the expression of 16 m6A regulators. The result showed that almost all m6A regulators were significantly correlated with the infiltration level of immune cells (**Figure S6F** and **Table S7**). The results indicated that these m6A regulators were dysregulated in the two clusters and among the infiltration subtypes (**Figures S5D, E**). Moreover, hazard ratios of survival associated with the high and low expression of m6A regulators in PAAD patients based on immune cell-enriched subgroups were also calculated using the Kaplan-Meier plotter database (**Figures S7A-G** and **Table S8**). Finally, we explored the expression level of 16 m6A genes in different cell types at the single-cell level across two datasets, which indicate all m6A regulators were expressed in immune cells (**Figure 3F** and **Table S9**).

### Identified Consensus Clustering Subtype and Landscape of the Immune-Related Index

**Figure 4:**
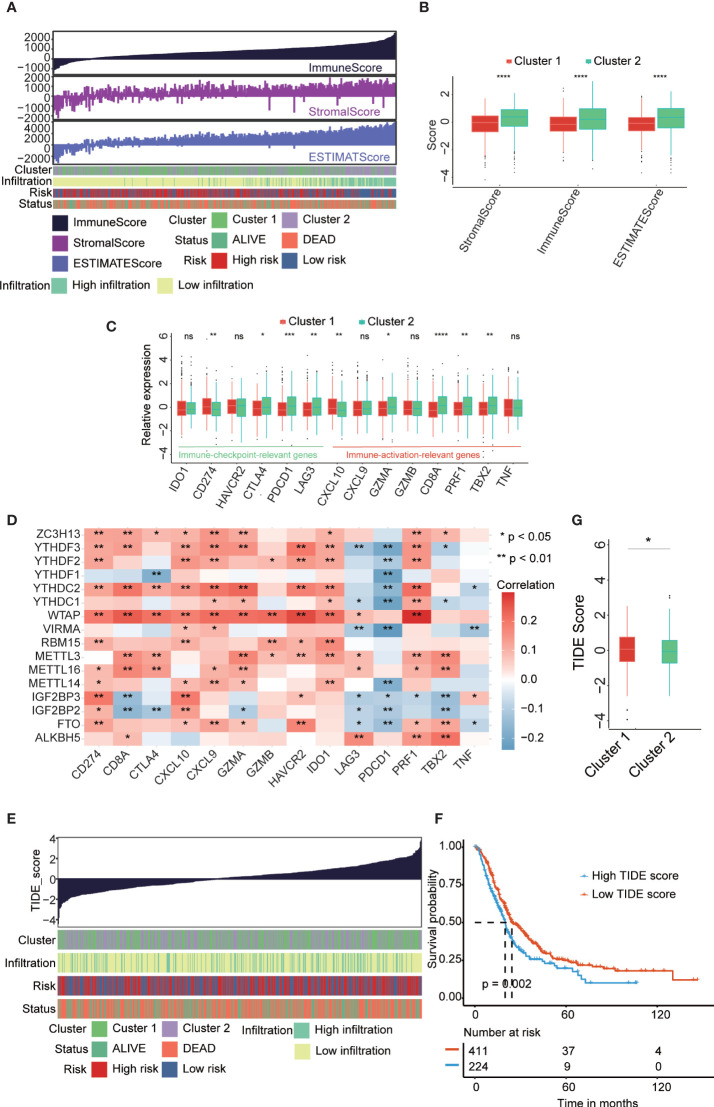
The correlation between clusters and tumor immune microenvironment. **(A)**. The associated landscape among immunescore, stromalscore, and estimatescore and molecular characteristics (cluster subtypes, Risk subtypes, immune infiltration subtypes, and survival status). Columns showed PAAD samples sorted by immunescore from low to high. **(B)** ImmuneScore, StromalScore, and estimatescore in two cluster subtypes. **(C)** Immune-activation-relevant genes and immune-checkpoint-relevant genes expressed in two cluster subtypes. **(D)** The correlation of the expression level of 16 m6A regulators and immune-activation-relevant and immune-checkpoint-relevant genes. **(E)**. The association overview between TIDE score and molecular characteristics (cluster subtypes, Risk subtypes, immune infiltration subtypes, and survival status). Columns showed PAAD samples sorted by TIDE score from low to high. **(F)** Kaplan-Meier curves for overall survival (OS) of all PAAD patients with high and low TIDE score. **(G)** Comparison of TIDE score in two cluster subtypes. *p < 0.05; **p < 0.01; ***p < 0.001; ****p < 0.0001; "ns" p > 0.05.

In order to understand which cluster of PAAD patients are more sensitive to immunotherapy, the relationship between clusters and the immune microenvironment in PAAD was further evaluated. We performed ESTIMATE algorithms to quantify the immune score and stromal score to further confirm the enrichment of immune-related cells in PAAD tumor tissues (**Figure 4A**). Patients in cluster 2 have higher ImmuneScore, StromalScore, and ESTIMATEScore than patients in cluster 1 (**Figure 4B**). Next, we analyzed the immune activity and tolerance condition of each cluster. CD274, CTLA4, HAVCR2, IDO1, LAG3, and PDCD1 were selected as immune checkpoint-relevant signatures, and CD8A, CXCL10, CXCL9, GZMA, GZMB, IFNG, PRF1, TBX2, and TNF were selected as immune activity-related signatures. We found that the immune checkpoint-relevant genes CTLA4, PDCD1 and LAG3 and the immune activity-related genes GZMA, CD8A, PRF1 and TBX2 were highly expressed in cluster 2 (**Figure 4C**). We also detected the correlations among the expression level of 16 m6A regulators and immune activation-relevant and immune checkpoint-relevant gene expression, which uncovered that most of these genes significantly correlated with immune-related genes (**Figure 4D** and **Table S10**). Based on the above results, we suspect that m6A regulators are associated with ICB therapy in patients with PAAD. A higher TIDE score suggested that tumor cells are more likely to escape immune surveillance and have a lower response rate to ICI treatment (**Figure 4E**). We confirmed that patients with low TIDE scores had a longer OS than those with high TIDE scores (**Figure 4F**). Consistently, we confirmed that patients in cluster 2 had a lower TIDE score than patients in cluster 1 (**Figure 4G**). We also analyzed the correlations among immune cell infiltration, TIDE score, immune score, and immune-related genes in the PAAD cohort (**Figures S8A-D**).

### Construction and Validation of the Prognostic Signature of m6A Regulators in the PAAD Cohort

**Figure 5:**
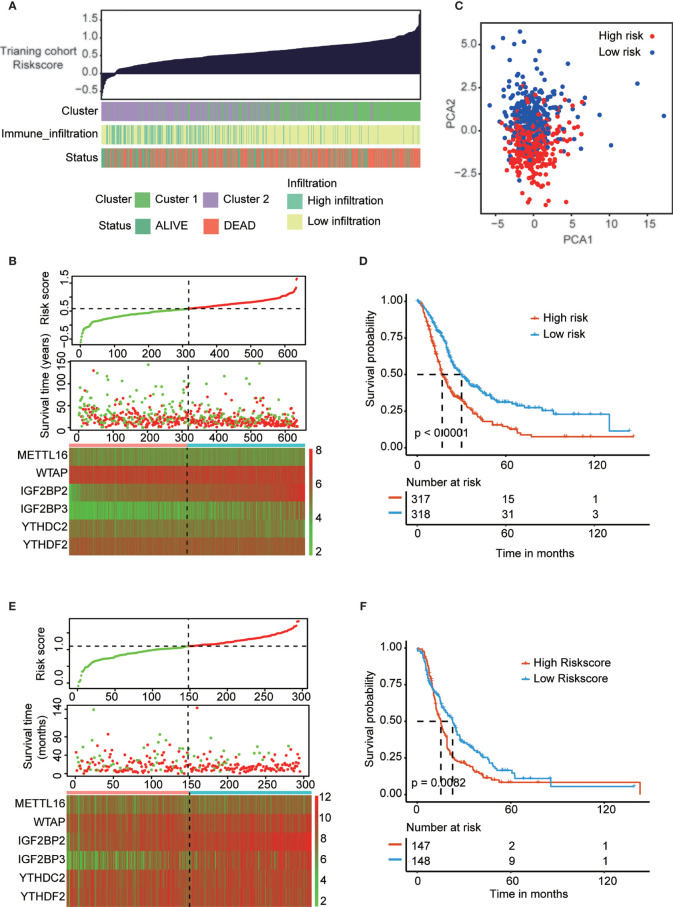
Construction and validation of prognostic signature of m6A regulators in PAAD cohort. **(A)** The associational landscape among risk score and molecular characteristics (cluster subtypes, immune infiltration subtypes, and survival status). Columns showed PAAD samples sorted by risk score from low to high. **(C)** Principal component analysis confirmed the two risk groups, high risk (red) and low risk (blue). **(B, E)** Distribution of risk score, OS, and OS status and heatmap of the six prognostic m6A regulator signatures in the training cohort **(B)** and validation cohort **(E)**. **(D, F)** Kaplan-Meier curves of OS for patients with PAAD based on the risk score in the training cohort **(D)** and validation cohort **(F)**.

All above results indicated that PAAD patients in cluster 2 are more suitable for immunotherapy. But the problem is that cluster groups will be difficult to be used in the real-world, thus we construct risk-score to provide a quantitative method for clinical application. To precisely predict the clinical outcome of m6A regulators in PAAD patients, we performed least absolute shrinkage and selection operator (LASSO) regression analysis based on the expression of 16 m6A regulators in the reorganized training cohort. Finally, we identified six m6A regulators, namely, METTL16, WTAP, IGF2BP2, IGF2BP3, YTHDC2 and YTHDF2 (**Figures S9A, B**). The risk scores were calculated using the coefficients obtained by the LASSO algorithm. The association landscape among the risk score and molecular characteristics (cluster subtypes, immune infiltration subtypes, and survival status) are shown in **Figure 5A**. And the PCA analysis confirmed significant difference in two risk subgroups (**Figure 5C**). The distributions of the risk score, OS, OS status and heatmap of the six prognostic m6A regulator signatures in the training cohort and validation cohort are shown in **Figures 5B, E**. Then, we explored the OS of PAAD patients based on the risk score in the training cohort (p<0.001) and validation cohort (p<0.01) (**Figures 5D, F**). The results indicated that patients in the low-risk group had a longer OS than patients in the high-risk group. To evaluate the prognostic accuracy of these 6 m6A regulators, we generated receiver operating characteristic curves at 1, 3, and 5 years in the PAAD training cohort (0.676, 0.662 and 0.665, respectively) and validation cohort (0.784, 0.768 and 0.756, respectively) (**Figures S9C, D**). The results validated the predictive advantage of the established risk model. Interestingly, cluster subtypes were significantly correlated with risk groups (p<0.001, **Figure S9E**), thus we suspect risk model can replace clusters subtypes to provide a quantitative method for clinical application.

### The Risk Model Was Associated with the PAAD Immune Microenvironment

**Figure 6:**
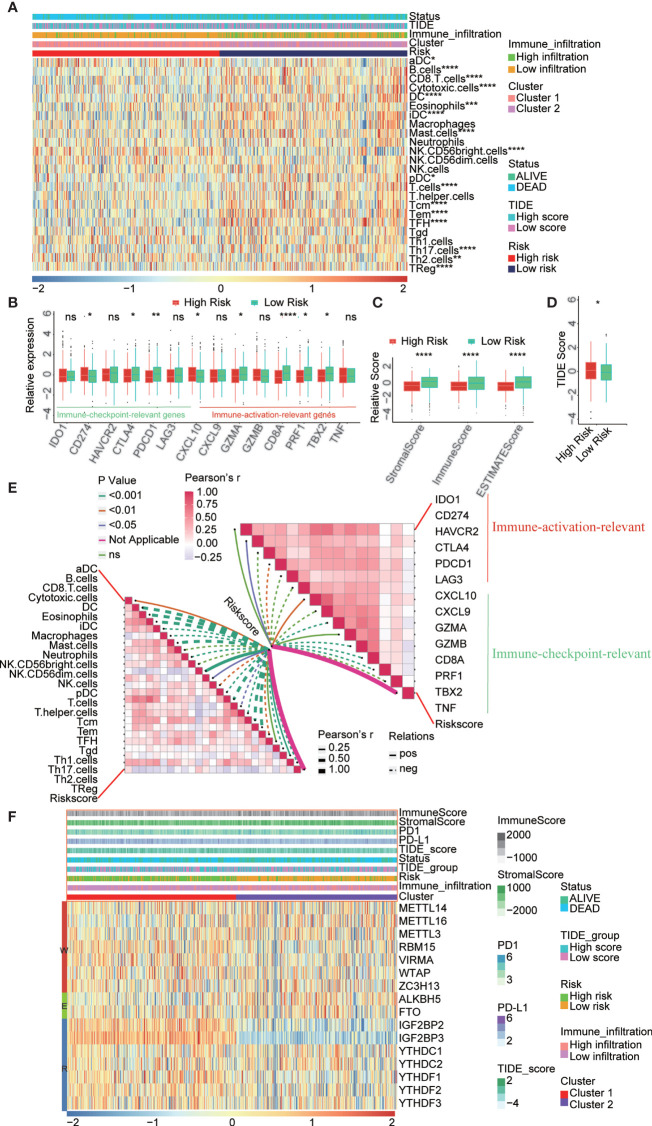
Correlation between risk score and immune sensitivity. **(A)** The heatmap showed the comparison of fraction of tumor-infiltrating immune cells in two risk subtypes. **(B)** ImmuneScore and StromalScore in high and low risk subtypes. **(C)** Immune-activation-relevant genes and immune-checkpoint-relevant genes expressed in high and low risk subtypes. **(D)** TIDE score in high and low risk subtypes. **(E)** The correlation of the risk score and infiltrating levels of tumor-infiltrating immune cells and immune-activation-relevant genes and immune-checkpoint-relevant genes expression. **(F)** The association landscape among 16 m6A regulators expression and known clinical features and molecular characteristics (ImmuneScore, StromalScore, PD1 expression, PD-L1 expression, TIDE score, cluster subtypes, immune infiltration subtypes, and survival status). *p < 0.05; **p < 0.01; ***p < 0.001; ****p < 0.0001;"ns" p > 0.05.

To verify the role of risk model in immunotherapy, we analyze the correlation between risk score and multiple immune-related indicators. The risk score was positively correlated with 109 immune-related genes and negatively correlated with 132 immune-related genes (**Figure S10A** and **Table S11**). To confirm that the risk model was associated with the PAAD immune microenvironment, we assessed the immune cell infiltration level between high- and low-risk score subtypes. The results revealed that most immune cells were highly enriched in patients with low-risk scores group (**Figure 6A**). Moreover, we assessed the correlation between the risk score and tumor-infiltrating immune cells (**Figure S10B** and **Table S12**). Additionally, immune activation-relevant gene and immune checkpoint-relevant gene expression was analyzed in high- and low-risk subtypes. We found that CTLA4, PDCD1, GZMA, CD8A, PRF2, and TBX2 were highly expressed in the low-risk subtype when compared to high-risk subtype (**Figure 6B**). Consistently, the low-risk subtype had a lower TIDE score and a higher ImmuneScore and StromalScore, indicating that patients in the low-risk subtype have more immune cell infiltration and higher sensitivity to immune checkpoint inhibitor treatment (**Figures 6C, D**). The correlation of the risk score and tumor-infiltrating immune cells, immune activation-relevant gene expression, and immune checkpoint-relevant gene expression was also conducted to further validate that the risk model was significantly correlated with the immune response (**Figure 6E**). The results uncovered that risk score was negatively correlated with most immune-related genes expression and immune cells infiltration levels. The correlation of each immune cell type in the high- and low-risk subtypes was also consistent with clusters, especially the cytotoxic cells and T cells have strongly positive correlation (**Figure S10C**). Finally, the associations among m6A regulator expression and known clinical features and molecular characteristics (immune score, stromal score, PD-1 expression, PD-L1 expression, TIDE score, cluster subtypes, immune infiltration subtypes, and survival status) are shown in **Figure 6F**. The correlation between the risk score and the expression of 16 m6A regulators is shown in **Figure S10D**.

### The Role of the Risk Score Model in the Prediction of Immunotherapeutic Benefits

**Figure 7:**
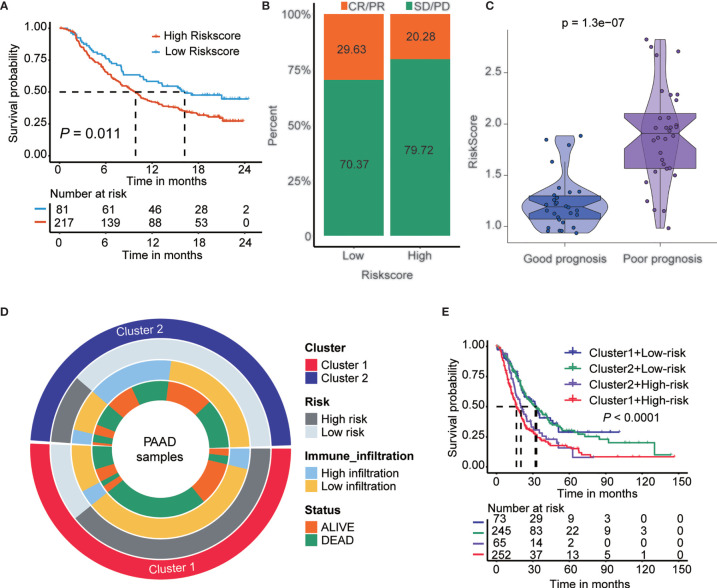
The role of risk score in the prediction of immunotherapeutic benefits. **(A)** Kaplan-Meier curves for patients with high and low risk score in the IMvigor210 cohort. **(B)** Rate of clinical response (complete response [CR]/partial response [PR] and stable disease [SD]/progressive disease [PD] to anti-PD-L1 immunotherapy in high and low risk score subgroups in the IMvigor210 cohort). **(C)** Violin plots depicted the differences in risk score in good prognosis and poor prognosis’ groups in the GSE148476 cohort. p < 0.001. **(D)** Alluvial diagram of m6A clusters distribution in groups with different clusters, risk score, immune infiltration, and survival outcomes. From the outside to the inside, each ring represents Cluster, risk group, immune infiltration, and survival status, respectively. **(E)** Kaplan-Meier curves for patients in the training cohort stratified by groups with different m6A gene clusters and risk score.

A lower TIDE score in the low-risk subtype indicated higher sensitivity to immune checkpoint inhibitor (ICI) treatment. Next, we examined the utility of the risk score in speculating the therapeutic benefit in patients. We downloaded the IMvigor210 cohort in which patients received anti-PD-L1 immunotherapy. We divided the patients into high- and low-risk score subtypes and analyzed their prognosis. The results showed that patients with low-risk scores had a longer OS (p<0.05) than patients with high-risk scores (**Figure 7A**). Next, we evaluated the rate of clinical response (complete response [CR]/partial response [PR] and stable disease [SD]/progressive disease [PD]) to anti-PD-L1 immunotherapy in the high- and low-risk score subgroups. The rate of CR/PR in the low-risk-score subtype was 29.63%, which was significantly higher than the 20.28% rate in the high-risk-score subtype (**Figure 7B**). The risk score was lower in group with good prognosis than those patients with poor prognosis in GSE148476 cohort (**Figure 7C**). Furthermore, we show an alluvial diagram of the PAAD patients distribution in groups with different clusters, risk groups, immune infiltration levels, and survival outcomes (**Figure 7D**). Additionally, we generated Kaplan-Meier curves for 4 groups of patients in the training cohort stratified by groups with different m6A gene clusters and risk scores. The results showed that patients with PAAD in cluster1-high-risk group have the worst prognosis (**Figure 7E**).

### The Underlying Mechanism and Pathways in the Risk Score Model

The risk score model is a great tool for predicting the therapeutic benefit in PAAD patients. However, the underlying mechanism and pathways remain to be fully explored. The cancer immunity cycle reflects the anticancer immune response and comprises seven steps (**Table S13**) (15): release of cancer cell antigens (step 1), cancer antigen presentation (step 2), priming and activation (step 3), trafficking of immune cells to tumors (step 4), infiltration of immune cells into tumors (step 5), recognition of cancer cells by T cells (step 6), and killing of cancer cells (step 7).

The activity of the cancer immunity cycle is a direct and comprehensive function of the chemokine subsystem and other immunomodulators (15, 16). As shown in **Figure S10A**, in the high-risk group, the release of cancer cell antigens increased during the cycle (step 1) but initiation and activation (step 3) and immune cell transport to tumors (step 4) (B cell recruitment, CD4 T cell recruitment, macrophage recruitment, T cell recruitment, Th2 cell recruitment, Th22 cell recruitment, DC recruitment and TH17 recruitment) decreased. The inhibition of these steps may reduce the penetration level of effector tumor-infiltrating immune cells in the tumor microenvironment. In the high-risk group, the activity of killing cancer cells (step 7) was downregulated.

**Figure 8:**
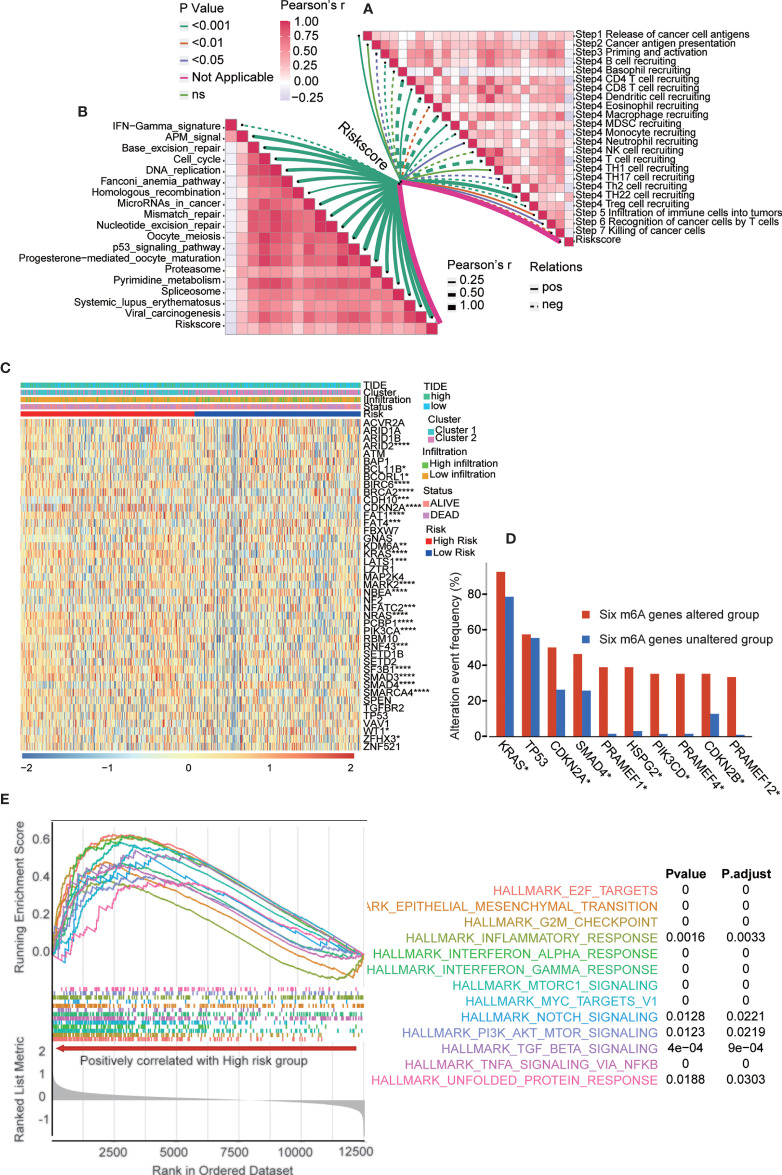
The underlying mechanism and pathways in the risk score model. **(A)** Correlation between risk score and the steps of the cancer immunity cycle. **(B)** Correlation between risk score and the enrichment score of immunotherapy-predicted pathways. **(C)** PAAD-specific cancer driver genes expressed in groups with different m6A gene clusters, immune infiltration, risk score, TIDE score, and survival status. The statistics results presented in the picture was analyzed between high and low risk subgroups. **(D)** The most altered genes frequency in six m6A genes altered and unaltered group. **(E)** GSEA showed multiple cancer-related signaling pathway are positively enriched in high-risk group. *p < 0.05; **p < 0.01; ***p < 0.001; ****p < 0.0001.

We also analyzed the correlation between the risk score and the steps of the cancer immunity cycle (**Figure 8A** and **Table S14**). The results showed that the risk score negatively modulated CD4+ T cell, dendritic cell, macrophage, and T cell recruitment. The present results helped to identify m6A regulator target immune cells. Additionally, we examined the correlation between the risk score and the enrichment score of immunotherapy-predicted pathways. The results revealed that the risk score positively correlated with the enrichment fraction of most immunotherapy-related pathways (**Figure 8B** and **Tables S15, 16**). Furthermore, we analyzed the PAAD-specific cancer driver genes expressed in groups with different m6A gene clusters, immune infiltration levels, risk scores, TIDE scores, and survival statuses (**Figure 8C**). The results showed that most PAAD-specific cancer driver genes were significantly upregulated in high-risk group. More specifically, we examined the frequencies of the most altered genes in the six m6A gene-altered and gene-unaltered groups. The results showed that KRAS, CDKN2A, SMAD4, PRAMEF1, HSPG2, PIK3CD, PRAMEF4, CDKN2B and PRAMEF12 had higher alteration event frequencies in the six m6A gene-altered group (**Figure 8D**). Next, we performed gene set enrichment analysis (GSEA) in the two risk subtypes (**Figure 8E**). GSEA results showed that multiple carcinogenic-related signaling pathways, including interferon-alpha-response, interferon-gamma-response, and inflammatory-response and other immune related signaling pathways, were positively enriched in the high-risk group. Moreover, gene set variation analysis (GSVA) was performed between the high- and low-risk groups in the validation cohort (**Figure S11**). TNF-α signaling *via* NF-κB, interferon-α signaling, interferon-γ signaling, epithelial-mesenchymal transition, MYC targets, mTOR signaling and many others were the shared pathways in these two analyses.

### Validation of the Expression of m6A Regulators in PAAD Patient Tissues and Cell Lines

**Figure 9:**
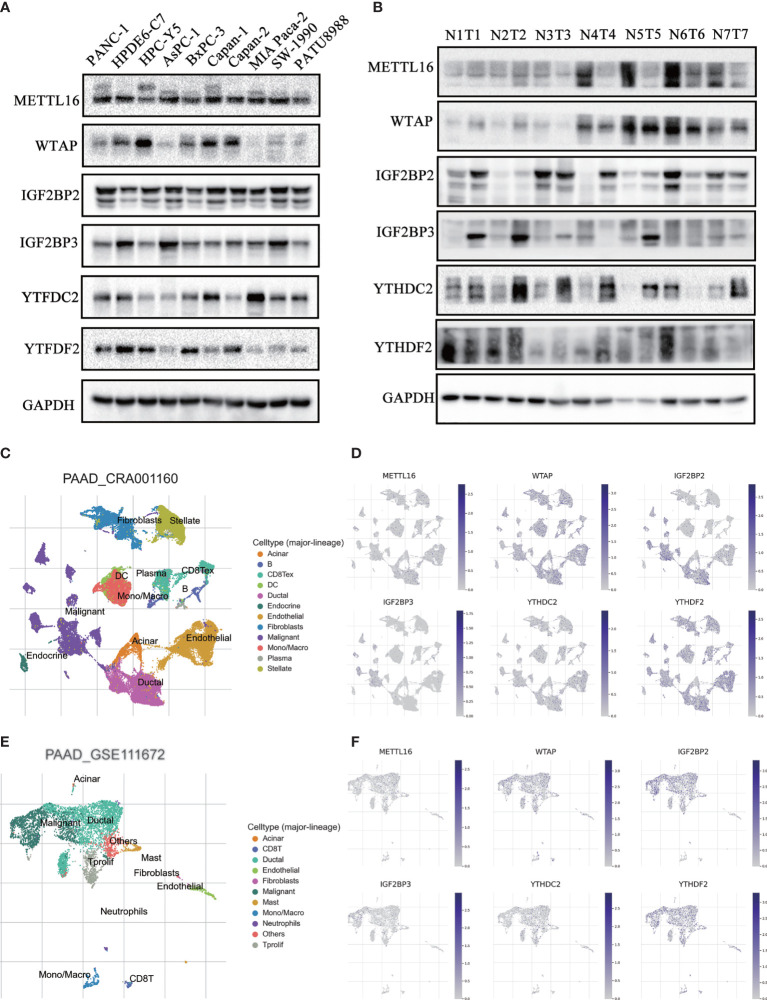
Validation of the expression of m6A regulators in PAAD patient tissues and cell lines. **(A, B)** The protein expression level of six risk-score-gene in PAAD cell lines and PAAD tissues and adjacent tissues. **(C, D)** UMAP visualization of dataset PAAD_CRA001160 and PAAD_GSE111672, respectively. **(E, F)** The distribution of six risk-score-genes expression in different cell types in PAAD at single cell level.

**Figure 10:**
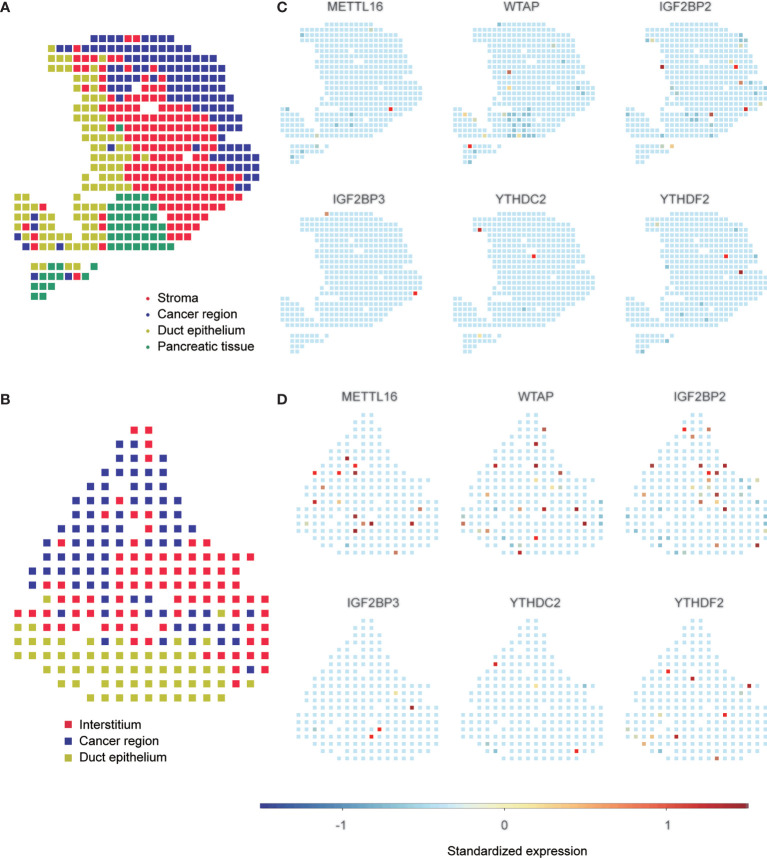
Spatial transcriptome analysis of m6A regulators. A, B, Clustering of the PDAC-A **(A)** and PDAC-B **(B)** spatial transcriptome spots. Colors represent different clustering. C, D, Standardized expression levels of m6A regulators in PDAC-A **(C)** and PDAC-B **(D)** datasets in spatial transcriptome.

To confirm the expression pattern of these six m6A regulators, we examined the mRNA and protein expression of these regulators in PAAD cell lines and tissues (**Figures 9A, B** and **Figures S12**, **S13**). The expression of METTL16 and WTAP was significantly decreased and the expression of IGFBP2 and IGF2BP3 was increased in most PAAD samples. We also showed UMAP visualization of infiltrating immune cells in the PAAD_CRA001160 and PAAD_GSE111672 datasets (**Figures 9C, E**). The distribution of the six risk score genes expression was also shown in different cell types in PAAD at the single-cell level (**Figures 9D, F**). WTAP, IGF2BP2, and YTHDF2 are mostly enriched in ductal cell and B cells. The recently developed spatial transcriptomics (ST) technology allows visualization of the distribution and characterize expression level of the transcriptome with spatial resolution over entire tissue sections (17). We get further revealed spatial patterns of these sis m6A regulators using GSE111672 dataset and methods as published paper mentioned (**Figure 10**) (18). Although the six genes are not highly frequent in either PAAD-A or -B, they give us the new insight to explore the underlying roles of m6A-related genes in PAAD.

## Discussion

Accumulating evidences have suggested that m6A modification plays a critical role in the cancer immune response and development (8, 13). As the most common posttranscriptional modification, m6A modification modulates the stability and translation processes of the targeted mRNA. The m6A methyltransferase METTL3 has been proven to promote PAAD progression, while the demethylase ALKBH5 inhibits PAAD tumorigenesis *via* posttranscriptional activation of PER1 and regulation of the Wnt pathway (13). Recently, m6A modification was implicated in the regulation of the immune microenvironment of multiple cancers, including PAAD (19). However, the expression pattern of m6A regulators in PAAD is still far from fully understood. Additionally, a methodology is urgently needed to quantify the comprehensive tumor immune microenvironment and to predict the immunotherapy benefits for PAAD patients.

Previously published pan-cancer studies have improved our understanding about the m6A regulators across kinds of tumors (20–22). However, the expression of m6A regulators in pan-cancer was analyzed only based on the data in TCGA database and the types of cancer is incomplete. In this study, TCGA and GTEx, oncomine and CCLE databases were used to analyze the expression level of m6A regulators in pan-cancer. Consistent with previous studies, different cancer types have specific expression patterns.

To accurately detect the expression of m6A regulators in PAAD, we integrated seven independent datasets, with a total of 930 patients with PAAD. Although M6A-related regulators have been reported in pancreatic cancer (23, 24). Geng et al. reported m6A-related genes and m6A RNA methylation regulators in PAAD and their prognostic performance based on TCGA database (25). Meng et. al (14) compared PAAD groups with/without genetic alteration of m6A regulators, identified differentially expressed genes to generate a 16-mRNA signature score system through LASSO analysis. However, to the best of our knowledge, this is the largest sample size of PAAD with clinical information to comprehensively analyze the association between immune microenvironment and m6A regulators. The results indicated that METTL3, IGF2BP2, IGF2BP3, YTHDC1, FTO and YTHDF1 were highly expressed, while METTL16, RBM15, ZC3H13 and ALKBH5 were downregulated in PAAD. Moreover, these regulators interacted with each other, indicating that m6A regulators operate together to regulate tumor progression. Genetic alteration is believed to play a critical role in modulating gene expression (26). To determine the underlying mechanism for the unique expression pattern of m6A regulators in PAAD, we analyzed the genetic alterations in these 16 target genes. Amplification and deep deletion seem to be the most common alterations. Copy number variations (CNVs) are also an alternative explanation for the specific expression pattern (27).

Although m6A modification has been reported to be involved in the immune response and microenvironment, it remains to be fully explored (28). By consensus clustering for m6A regulators, the integrated dataset was divided into cluster 1 and cluster 2. Briefly, cluster 2 showed lower m6A regulator expression and had better survival. Similarly, cluster 2 had a higher immune cell infiltration. In detail, cluster 2 had a higher density of B cells, CD8+ T cells, cytotoxic T cells, dendritic cells and natural killer cells, which have been proven to play an antitumor role. Moreover, immune-related signatures and immune-related scores, including the immune score, stromal score, and TIDE score, were analyzed in these two clusters. Consistently, cluster 2 showed higher expression of immune-related signatures, higher immune scores, higher stromal scores, and lower TIDE scores. These results indicated that m6A regulators were related to PAAD prognosis and that the immune microenvironment was a potential mechanism accounting for the phenotype. Additionally, these results indicated that PAAD patients in cluster 2 were more sensitive to immunotherapy. However, it’s difficult to apply the cluster groups to the clinical use. It’s urgent to provide a quantitative method for clinical application.

Considering the individual heterogeneity of the immune microenvironment, an individual-based model based on specific biomarkers is urgently needed. As shown, m6A regulators were tightly related to the immune landscape of PAAD patients, indicating that m6A regulators may be a promising indicator for constructing a prediction model. By performing least absolute shrinkage and selection operator (LASSO) regression analysis, six m6A regulators, namely, METTL16, WTAP, IGF2BP2, IGF2BP3, YTHDC2 and YTHDF2, were utilized to construct the risk model. The risk score obtained from the risk model effectively stratified the patients into high-risk and low-risk subgroups. Patients in the low-risk group had better survival. Consistently, the low-risk group showed a higher density of immune cell infiltration, higher expression of immune-related signatures, a higher immune score and stromal score and a lower TIDE score. These results all proved that the risk score is a promising indicator to assess the immune microenvironment of PAAD patients. Expression of immune checkpoint signatures, such as PD-L1 or the tumor mutation burden (TMB), are potential indicators to predict the immunotherapy response (29, 30). In the present study, we assessed the patients receiving immunotherapy in the IMvigor210 cohort and found that the risk score was negatively related to the immunotherapy response. The patients responding to immunotherapy had lower risk scores, indicating that the risk score had a predictive role.

The activity of the cancer immunity cycle is a direct and comprehensive function of the chemokine subsystem and other immunomodulators. We analyzed the relationship between the risk score and the cycle steps. Interestingly, we found that step 1 (the release of cancer antigens) was enhanced in the high-risk subgroup compared with the low-risk subgroup, which seems counterintuitive to our predictions. Malignant cancer cells have a higher metabolic rate and demand more nutrients and oxygen (31, 32). As a result, these cells will release more metabolites. Furthermore, a hypoxic environment exists inside tumors that may cause necrosis of cancer cells (33). These results help to account for the higher release of cancer antigen in the high-risk subgroup. Although the release of cancer antigens increased, there was no significant difference in step 2 (cancer antigen presentation) between these two subgroups. Furthermore, the priming, activation and recruitment of infiltrating immune cells were decreased in the high-risk subgroup compared with the low-risk group. These results indicated that the quantity and quality of infiltrating immune cells seem to be critical factors influencing the immune response of PAAD. It is of little significance if the higher antigen release fails to activate and recruit exhausted immune cells (34). Measures to increase immune cell infiltration seem to be a potential strategy to improve the immune response and prolong the prognosis of PAAD patients (35).

Although the role of the risk score model was identified, it is important to determine the underlying pathways and molecular characteristics. Here, we identified that KRAS, CDKN2A, SMAD4, PRAMEF1, HSPG2, PIK3CD, PRAMEF4, CDKN2B and PRAMEF12 had higher alteration event frequencies in six m6A genes in the altered group. It has been reported that KRAS promotes PAAD immune evasion and that SMAD4 is involved in the TGF-β signaling pathway, which influences the immune response (36, 37). Our results were consistent with these reports. GSEA and GSVA were used to uncover the malignant functional features in risk subgroups. The results showed that TNF-α signaling *via* NF-κB, interferon-α signaling, interferon-γ signaling, epithelial-mesenchymal transition, MYC targets, and mTOR signaling were the potential downstream pathways of m6A regulators. Previous studies have proven that these pathways are involved in immune microenvironment regulation (38, 39).

Although the risk score model can be utilized to quantify the PAAD immune microenvironment and speculate about immunotherapy benefits, not all patients with low-risk scores positively responded to immunotherapy. More clinicopathological scores or signatures should be incorporated into this risk model to improve its accuracy. In pan-cancer, risk score genes are positively related to PD-L1 expression, which has been proven to act as an immunotherapy response indicator, indicating that the risk model may also work in other cancer types. However, more research work is needed to examine and promote this model in other cancer types.

In summary, we comprehensively analyzed the immune microenvironment, the expression pattern and functional role of m6A regulators, and the association among these factors in an integrated dataset of 930 PAAD patients with clinical information. We confirmed that m6A regulators were significantly related to the prognosis and immune landscape of PAAD patients. Moreover, we constructed a risk score model to quantify the immune microenvironment and predict the immunotherapy response. Our present study not only offered a novel perspective to understand the relationship between m6A regulators and the immune microenvironment but also provided potential targets for PAAD diagnosis, prognosis, and therapy.

## Materials and Methods

### Collection of PAAD Datasets and Preprocessing

**Figure 11:**
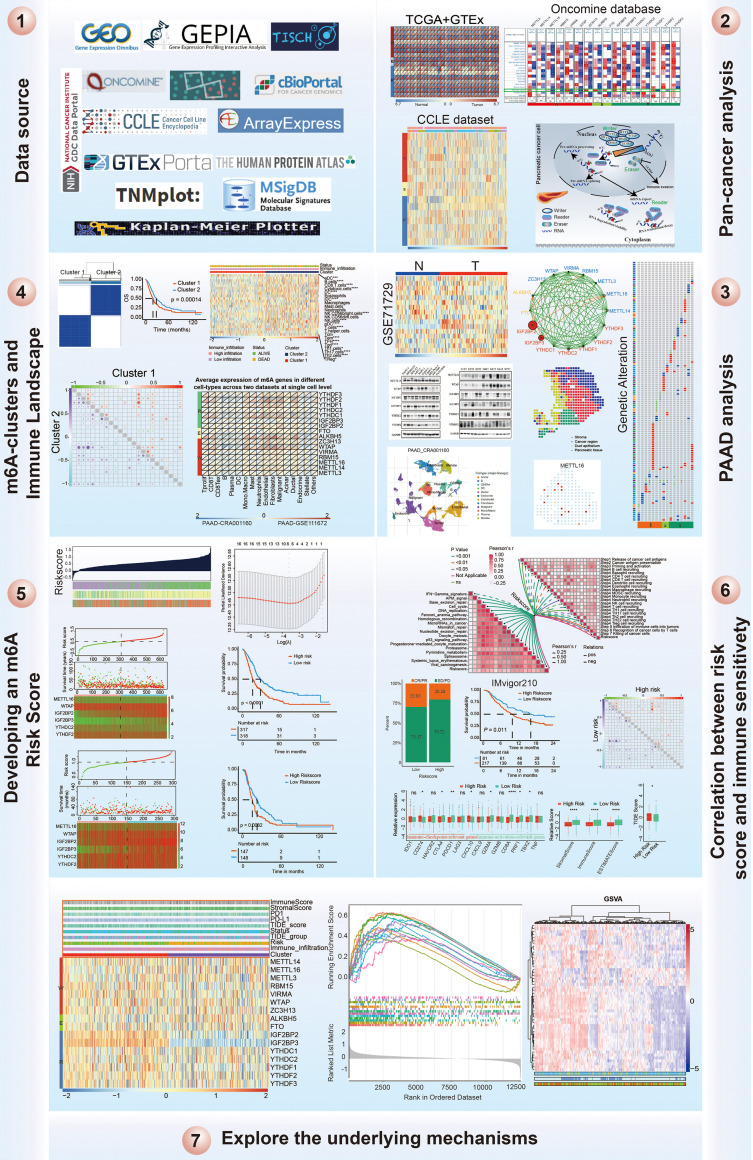
Workflow of this study design.

The workflow of this study is shown in **Figure 11**. Publicly available gene expression data and clinical annotations of the datasets were obtained from the Gene Expression Omnibus (GEO), The Cancer Genome Atlas (TCGA), International Cancer Genome Consortium (ICGC), and ArrayExpress databases. Patients without survival information were removed from further analysis. For the TCGA-PAAD cohort, RNA sequencing data (fragments per kilobase of transcript per million mapped reads [FPKM] values) were downloaded *via* the TCGAbiolinks (40) package based on R software. Then, FPKM values were transformed into transcripts per kilobase million (TPM) values that were more similar to those results from microarrays. Batch effect results from nonbiological technical biases were corrected using the ComBat method from the “SVA” package (41). In total, we gathered the GSE28735, GSE57495, GSE62452, MTAB-6134, and TCGA-PAAD datasets, including 635 patients as the training cohort for further analysis. We gathered the ICGC-AU and ICGC-CA datasets, including 295 patients as the validation cohort for further analysis. Finally, a total of 930 patients with PAAD with clinical information were included in this study, and the sample data used in this work are summarized in **Table S1**.

### m6A RNA Methylation Regulator Detection

According to previously published literature, 17 m6A regulators were collected (42, 43). A total of 16 m6A regulators were identified based on the available expression of the batch effect PAAD dataset. Furthermore, the mRNA expression of 16 m6A regulators across all cancer types was obtained from the gene expression profiling interactive analysis (GEPIA) (44) and Oncomine (45) databases, and the expression of these genes in cancer cell lines was obtained from the Human Cancer Cell Line Encyclopedia (CCLE) database (46). The differential expression of these genes in pancreatic cancer tissues versus normal tissues was also determined based on the GSE71729 dataset. The protein expression of m6A genes in PAAD and normal tissues was obtained from the Human Protein Atlas (HPA) database (47). The frequency of m6A regulator alterations (amplification, deep deletion, missense mutations, and so on) in PAAD patients was evaluated using the cBioPortal database, which is an interactive user-friendly platform that provides large-scale cancer genomics data for visualization and analysis (http://www.cbioportal.org/) (48, 49).

### Consensus Clustering for m6A Regulators

To functionally elucidate the biological characteristics of the m6A regulators in PAAD, the unsupervised clustering “ConsensusClusterPlus” package was utilized to classify the PAAD patients into different subtypes based on the expression of 16 m6A regulators and repeated 1000 times to ensure classification stability (50). The principal component analysis was performed using the “PCA” package in R software.

### Immune Cell Infiltration Estimated by Single-Sample Gene Set Enrichment Analysis and the Deconvolution Algorithm

The infiltration levels of 24 immune cell types in the tumor microenvironment were quantified by the ssGSEA method using the gsva package (51). Specific marker genes for each immune cell type were derived from published papers (52, 53). The deconvolution approach used in our study included 24 immune cells that are involved in immunity (54). Immune-related genes were obtained from recently published studies (55, 56). To facilitate further characterization, unsupervised clustering was applied to categorize the PAAD cohort into different subtypes based on immune cell infiltration levels.

### Quantification of the Immune Response Predictor: ESTIMATE and Tumor Immune Dysfunction and Exclusion

Immune checkpoint blockade (ICB) therapy effectively provides clinical benefits and helps the immune system recognize and attack cancer cells, but only a small portion of cancer patients respond to therapy (57). The immunoscore and stromal score for every PAAD patient were calculated using the ESTIMATE algorithm using the “estimate” package, which takes advantage of the unique properties of mRNA expression to infer tumor cellularity and tumor purity (58). Tumor tissues with abundant immune cell infiltration show higher immune scores and lower levels of tumor purity (59). The TIDE algorithm was used to model distinct tumor immune evasion mechanisms, and a higher TIDE score predicts tumor cells that are more likely to induce immune evasion, meaning a lower response rate to immunotherapy (57).

### Generation of the m6A Score

The prognostic risk signature of 16 m6A regulators was determined using least absolute shrinkage and selection operator (LASSO) regression analysis in the final PAAD training cohort (60). Signatures were screened by selecting the optimal penalty parameter λ correlated with the minimum 10-fold cross validation. The coefficient obtained from the LASSO regression algorithm was used to yield the following risk score equation: risk score = sum of coefficients*m6A regulator expression level. Furthermore, the PAAD patients were separated into the high-risk and low-risk groups, where the cutoff point was based on the median value of the risk score.

### Collection of Immunotherapy Data

The IMvigor210 dataset, which investigates the clinical significance of PD-L1 blockade treatment with atezolizumab in metastatic urothelial cancer, was freely obtained from http://research-pub.gene.com/IMvigor210CoreBiologies and analyzed to determine the predictive value of the m6A-related risk score, and a total of 298 patients from the IMvigor210 cohort were included in this work to predict the m6A-related risk score in immunotherapy (61). The dataset for chronic lymphocytic leukemia patients treated with avadomide alone or combination with immunotherapy was downloaded from GEO under accession number GSE148476.

### GSEA and GSVA

The potential mechanisms of high- and low-risk subtypes in PAAD were explored using gene set enrichment analysis (GSEA) and gene set variation analysis (GSVA). The hallmark gene set (h.all.v6.2.entrez.gmt) was obtained from the Molecular Signatures Database (MSigDB) using GSEA V3.0 (http://software.broadinstitute.org/gsea/msigdb/index.jsp) (62) and the clusterprofiler (63) package in R software. *P*<0.05 and a false discovery rate (FDR) < 0.05 were considered statistically significant. Moreover, the cancer immunity cycle and immunotherapy-predicted pathways were also explored based on previously published papers (15, 64).

### Cell Culture

Cell lines were obtained from Procell Life Science & Technology Company (Wuhan, China) and Cell resource center, Shanghai Academy of Biological Sciences, Chinese Academy of Sciences (Shanghai, China). AsPC-1, BxPC3, HPDE6-C7 and PATU8988 cells were cultured with 1640 media (SH30809.01B, HyClone, USA). MIA Paca2, HPC-Y5 and PANC-1 cells were cultured with DMEM media (SH30022.01, HyClone, USA). SW-1990 cells were cultured with L15 media (SH30525.01, HyClone, USA). Capan-1 cells were cultured with IMDM media (31980030, Thermo, USA). Capan-2 cells were cultured with McCoys 5A media (12330031, Thermo, USA). The medias were supplemented with 10% fetal bovine serum (FBS) (10100147, Gibco, USA) and 1% penicillin-streptomycin. The cell incubator was kept at 37℃ with 5% CO2.

### Western Blot

Pancreatic patient tissues and normal tissues were obtained from the Department of General Surgery at Tangdu Hospital of Fourth Military Medical University (Xi’an, Shaanxi, People’s Republic of China). All procedures were approved by the Research Ethics Committee of Tangdu Hospital, and written informed consent was obtained from all patients. The PAAD tissues and normal tissues were cut into small pieces and then suspended in lysis buffer (P0013C, Beyotime, Shanghai, China) on ice. The samples were separated by 10% sodium dodecyl sulfate-polyacrylamide gel electrophoresis and then transferred to polyvinylidene fluoride (PVDF) membranes (3010040001, Roche, USA). The membranes were blocked with 5% nonfat milk and then incubated with primary antibodies at 4°C overnight. Primary antibodies against METTL16 (A15894), WTAP (A14695), YTHDF2 (A15616), IGFBP2 (A2749), IGF2BP3 (A4444) and YTHDC2 (A15004) were purchased from Abclonal (Wuhan, China). The membranes were washed with PBS three times and incubated with secondary HRP antibody for 2 hours at room temperature. Protein expression was examined using ECL (ChemiDoc MP, Bio-Rad, USA).

### Real-Time qPCR

According to instruction of the manufacturer, total RNA was extracted from HPDE6-C7, MIA Paca2, Capan-2, SW1990, PATU8988 and PANC-1 cells using TRIzol^®^. cDNA was synthetized using an ETC811 Real-Time PCR Detection System (Dongshenglong, China). ChamQtm SYBR qPCR master mix was used to quantify of target gene mRNA. The PCR cycle was as follows: 95℃ for 30 sec, 40 cycles of 15 sec at 95°C and 60°C for 30 sec. The sequences of the primers used are listed below. METTL16-F: CTCTGACGTGTACTCTCCTAAGG; METTL16-R TACCAGCCATTCAAGGTTGCT; WTAP-F, CTTCCCAAGAAGGTTCGATTGA; WTAP-R, TCAGACTCTCTTAGGCCAGTTAC; YTHDF2-F, AGCCCCACTTCCTACCAGATG; YTHDF2-R, TGAGAACTGTTATTTCCCCATGC; IGFBP2-F, GACAATGGCGATGACCACTCA; IGFBP2-R, CAGCTCCTTCATACCCGACTT; IGF2BP3-F, TATATCGGAAACCTCAGCGAGA; IGF2BP3-R, GGACCGAGTGCTCAACTTCT; YTHDC2-F, AGGACATTCGCATTGATGAGG; YTHDC2-R, CTCTGGTCCCCGTATCGGA; GAPDH-F, CGGAGTCAACGGATTTGGTCGTAT; GAPDH-R, AGCCTTCTCCATGGTGGTGAAGAC. GAPDH was used as the internal control using the 2^-ΔΔCt^ method.

### Statistical Analysis

The statistical analyses in the present work were carried out using R-4.0.4. For quantitative data, statistical significance for normally distributed variables was estimated by Student’s t-tests, and nonnormally distributed variables were analyzed by the Wilcoxon rank-sum test. For comparisons of more than two groups, the Kruskal-Wallis test and one-way ANOVA were used as nonparametric and parametric methods, respectively. Survival analysis was generated by the Kaplan-Meier method and the Cox proportional hazards model to analyze the association between the factors and prognosis using the “survival” and “Survminer” packages. The surv-cutpoint function from the “survival” package was applied to divide the PAAD samples used into different subtypes. The predictive performance of the m6A risk signature for 1-, 3-, and 5-year OS was assessed by receiver operating characteristic (ROC) curves carried out using the “timeROC” package (65). Correlations between the individual expression of m6A regulators, risk scores, immune-related genes, and immune cell infiltration levels were evaluated with the Pearson correlation coefficient and performed using the Corrplot (66) package in R software. The principal coordinates analysis (PCoA) was performed using PERM-ANOVA test. All comparisons were two-sided with an alpha level of 0.05, and the Bonferroni method was performed to control the false discovery rate (FDR) for multiple hypothesis testing.

## Data Availability Statement

The original contributions presented in the study are included in the article/**Supplementary Material**. Further inquiries can be directed to the corresponding authors.

## Author Contributions

HS and XL conceived and designed the whole project. YG, RW, and JL drafted the manuscript. JS, CZ, PM, CY, LZ, SL, and DG collected the data YS, JM, and TZ analyzed the data and wrote the manuscript. All authors reviewed and approved the final manuscript.

## Funding

This study was supported by a grant from the National Natural Science Foundation of China to HS (No. 81372255) and to RW (No. 81902523).

## Conflict of Interest

The authors declare that the research was conducted in the absence of any commercial or financial relationships that could be construed as a potential conflict of interest.

## Publisher’s Note

All claims expressed in this article are solely those of the authors and do not necessarily represent those of their affiliated organizations, or those of the publisher, the editors and the reviewers. Any product that may be evaluated in this article, or claim that may be made by its manufacturer, is not guaranteed or endorsed by the publisher.
