# Supplementary material for: Comprehensive Analysis of m6A RNA Methylation Regulators and the Immune Microenvironment to Aid Immunotherapy in Pancreatic Cancer
Source: Front Immunol. 2021 Nov 5;12:769425. doi: 10.3389/fimmu.2021.769425 (PMC8602908; doi:10.3389/fimmu.2021.769425)
Supplement: Supplementary file 1 [file DataSheet_1.docx]

**Comprehensive Analysis of m6A RNA Methylation Regulators and the Immune Microenvironment to Aid Immunotherapy in Pancreatic Cancer**

Yongdong Guo^1#^, Ronglin Wang^1#^, Junqiang Li^1#^, Yang Song^1^, Jie Min^1^, Ting Zhao^1^, Lei Hua^1^, Jingjie Shi^1^, Chao Zhang^1^, Peixiang Ma^1^, Cheng Yang^1^, Liaoliao Zhu^1^, Dongxue Gan^1^, Shanshan Li^1^, Xiaonan Liu^2*^, Haichuan Su^1*^

1, Department of Oncology, Tangdu Hospital, Air Force Medical University

2, Department of Ambulatory Surgery Center, Xijing Hospital, Air Force Medical University

3, # These authors contribute equally to this paper

4, *Corresponding author,

Haichuan Su, Department of Oncology, Tangdu Hospital, Air Force Medical University, NO. 569 Xinsi Road, Xi’an 710038, Shaanxi, China. Tel: +86-29-84717681; E-mail: [suhc@fmmu.edu.cn](mailto:suhc@fmmu.edu.cn)

Xiaonan Liu, Department of Ambulatory Surgery Center, Xijing Hospital, Air Force Medical University, NO.127 Changle West Road, Xi’an 710032, Shaanxi, China. Tel: +86-15353589999; E-mail: [15353589999@163.com](mailto:15353589999@163.com)

**Supplementary**


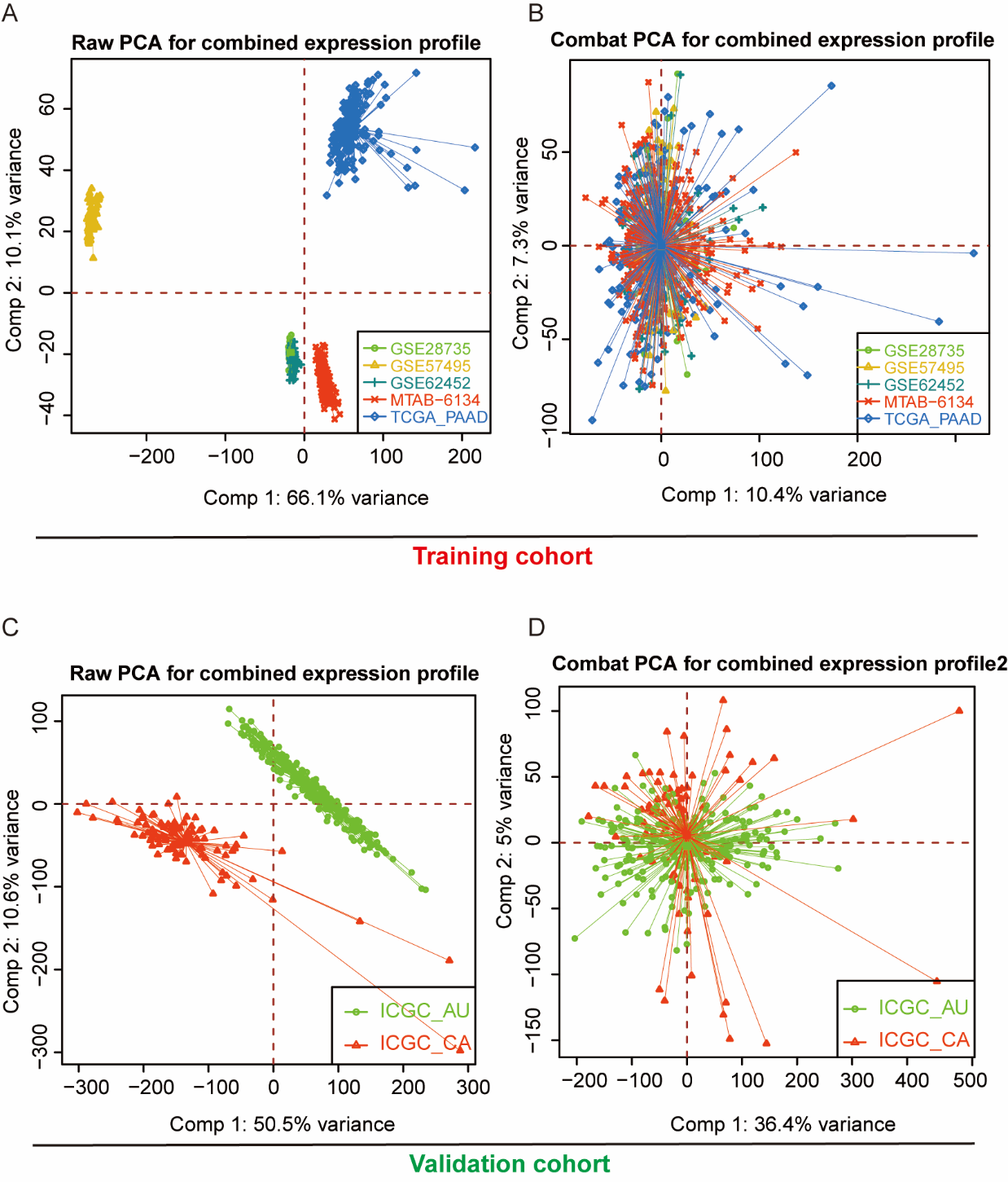


Figure S1. The PCA diagram of batch effect of the five datasets. The combat algorithm was applied to reduce the likelihood of batch effect from non-biological technical biases between seven datasets. (A) The PCA diagram without removing batch effect in training cohort. (B) The PCA diagram with removing batch effect in training cohort. (C) The PCA diagram without removing batch effect in validation cohort. (D) The PCA diagram with removing batch effect in validation cohort.


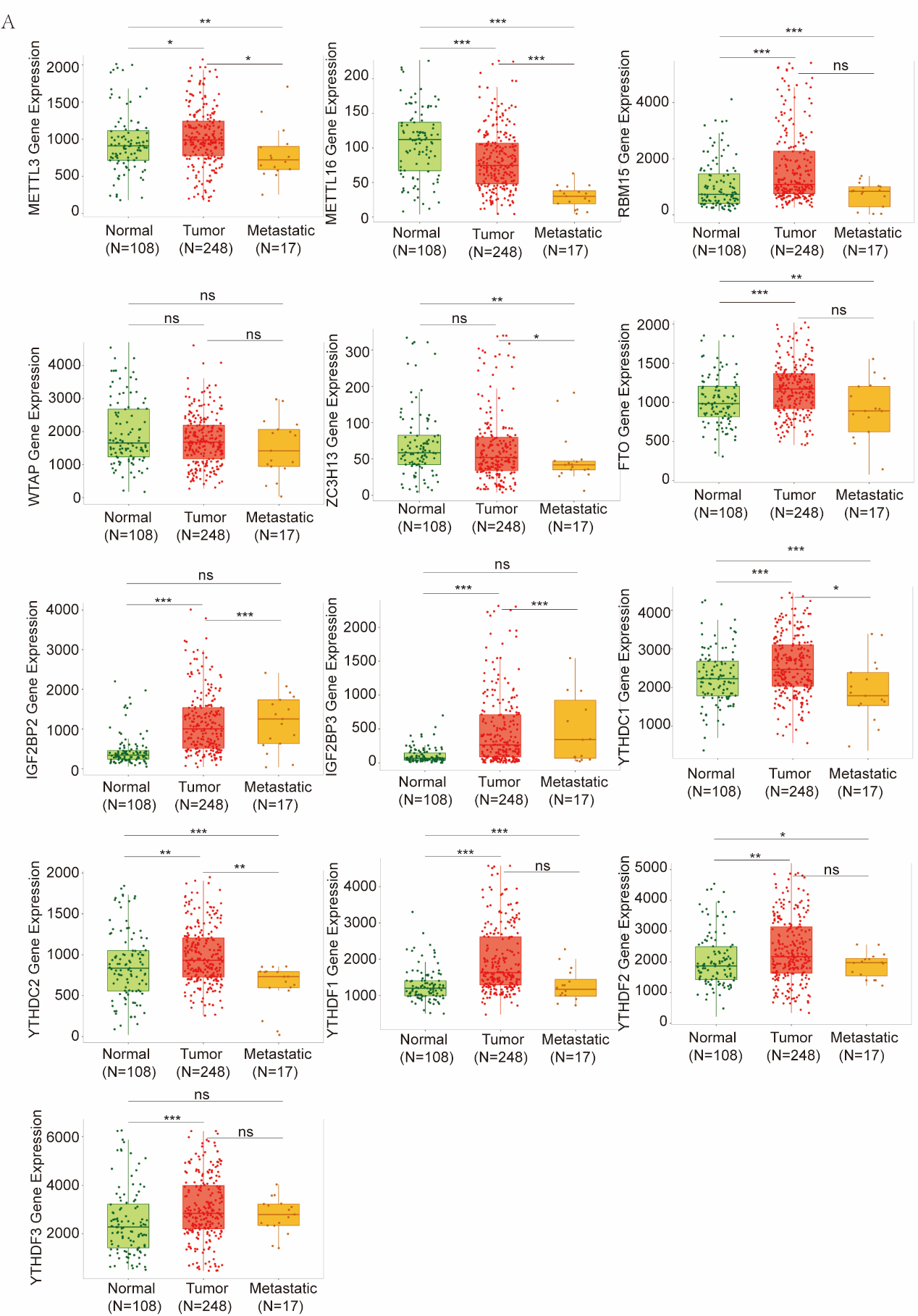


Figure S2. Boxplots of m6A genes expression level in PAAD when compared normal, tumor and metastatic tissues obtained from TNMplot database.


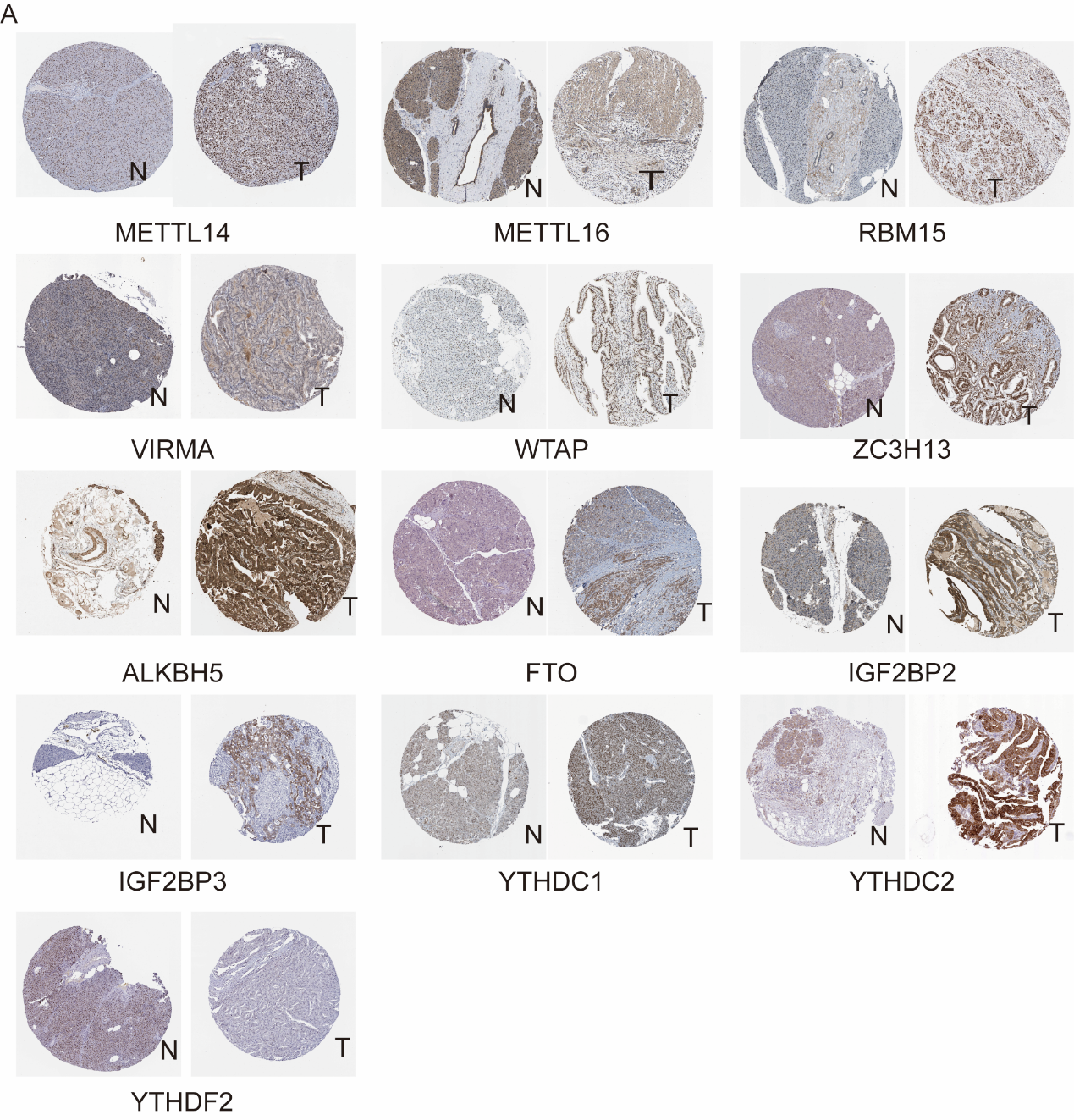


Figure S3. The protein expression of m6A genes in PAAD and normal tissues obtained from the human protein atlas (HPA) database.


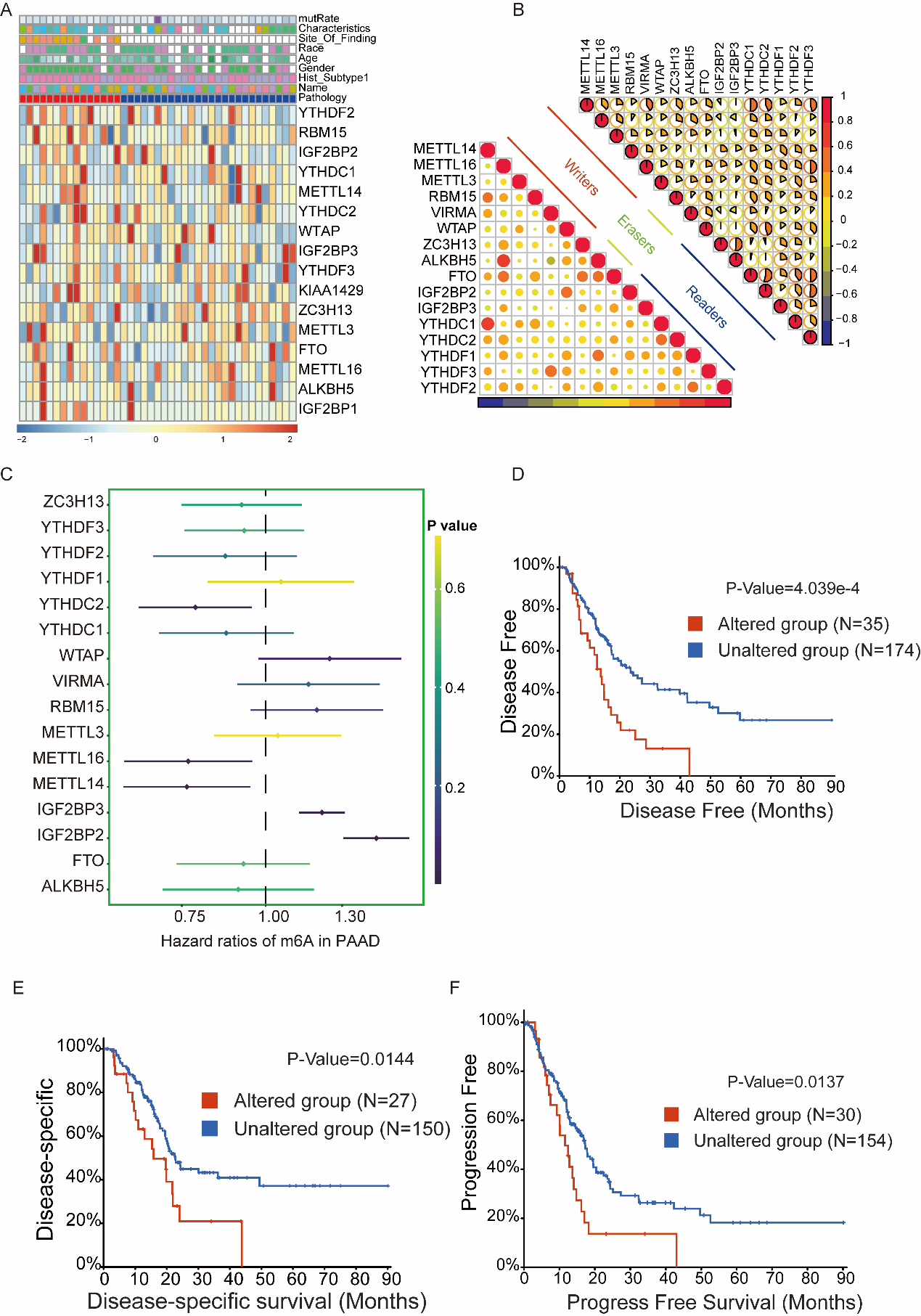


Figure S4. Expression and clinical relevance of m6A genes in PAAD. (A) Heatmap represents the relative expression of m6A regulators in PAAD cell lines. (B) The correlation heatmap of m6A regulators in PAAD cell lines and tissues. Left represents cell lines and right represents PAAD tissues. (C) The forest plot of m6A regulators clinical significance in PAAD. (D-F) Kaplan-Meier plots comparing DFS, DSS, and PFS in cases with and without 16 m6A regulators alterations in patients with PAAD.


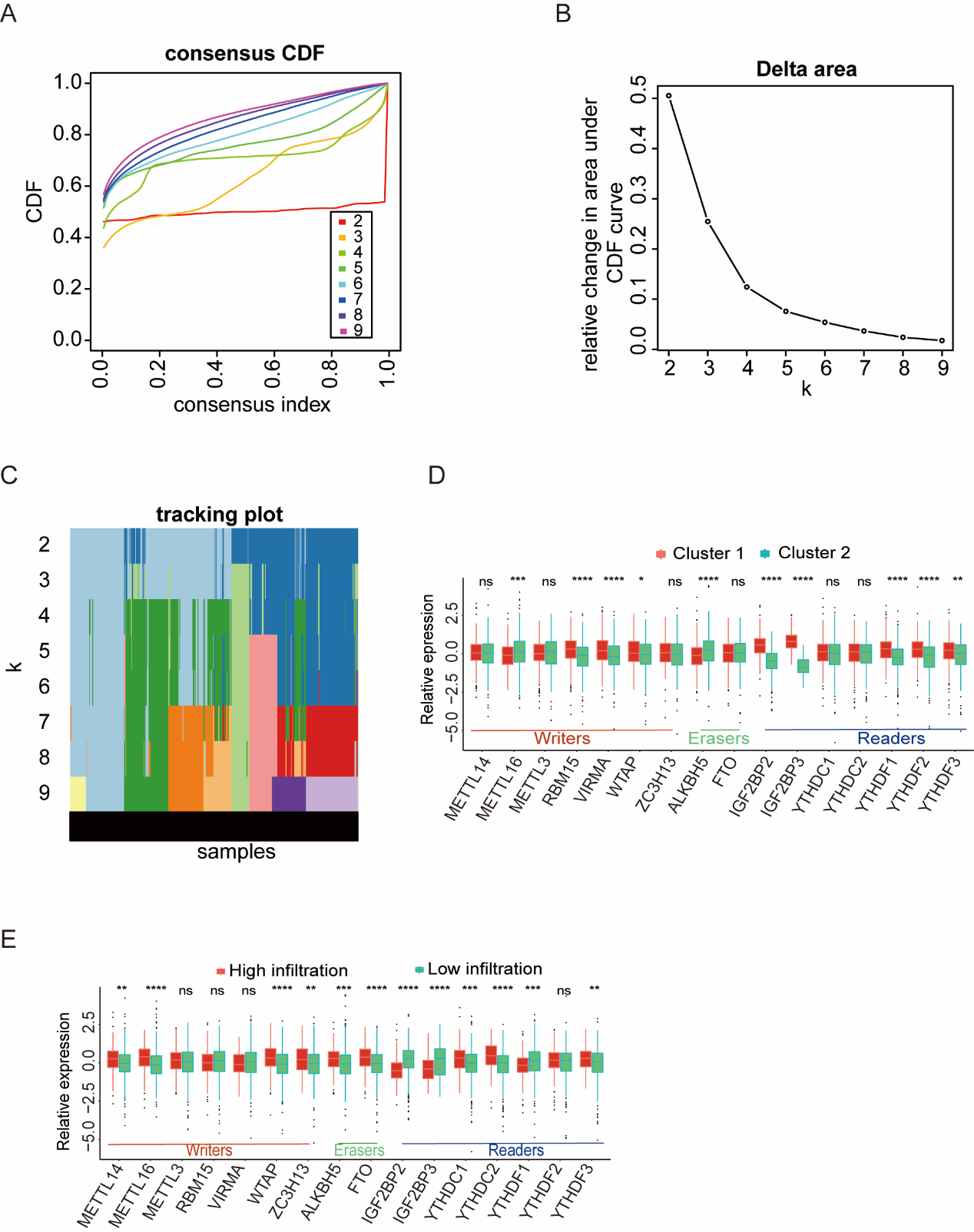


Figure S5. Consensus clusters by m6A regulators in PAAD cohort. (A) Consensus clustering cumulative distribution function (CDF) for k=2 to 9. (B) Relative change in area under the CDF curve for k=2 to 9. (C) Tracking plot for k=2 to 9. (D and E) The relative expression of m6A regulators in two clusters and two infiltration subtypes, respectively. *p<0.05; **p<0.01; ***p<0.001; ****p<0.0001.


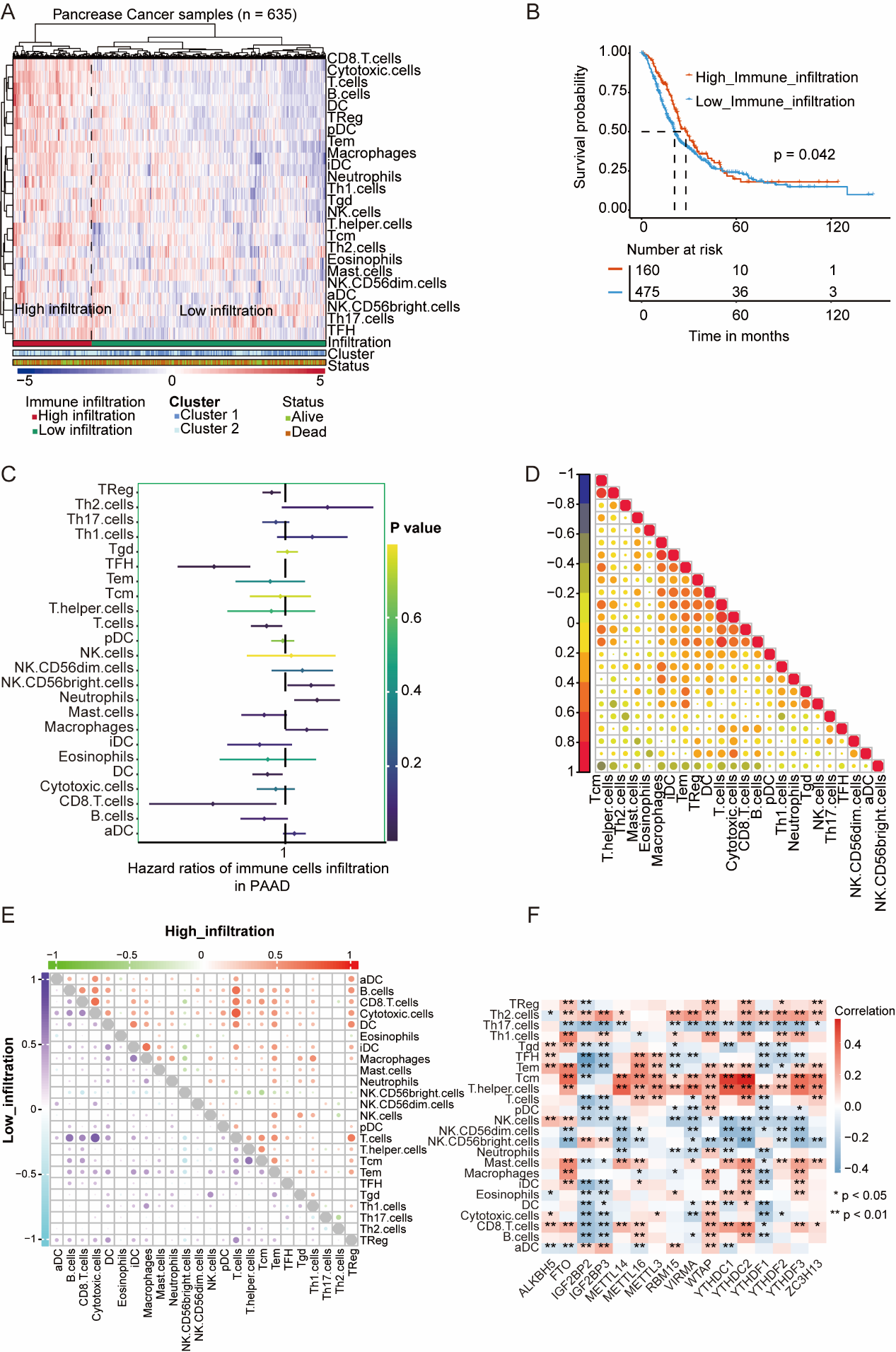


Figure S6. Immune infiltration landscape of 635-PAAD cohort. (A) Unsupervised clustering of tumor-infiltrating immune cells in 635-PAAD cohort. (B) Kaplan-Meier curves for overall survival (OS) of all PAAD patients with two immune infiltration subtypes. (C) The forest plot of tumor-infiltrating immune cells clinical significance in PAAD. (D) The correlation of the infiltrating levels of each immune cell in all PAAD patients. (E) The correlation of the infiltrating levels of tumor-infiltrating immune cells in high and low infiltration subtypes, respectively. Bubble size and color represents correlation coefficient r, don’t draw bubbles when p-value > 0.05. (F) The correlation of the infiltrating levels of tumor-infiltrating immune cells and 16 m6A regulators expression. *p<0.05; **p<0.01; ***p<0.001; ****p<0.0001.


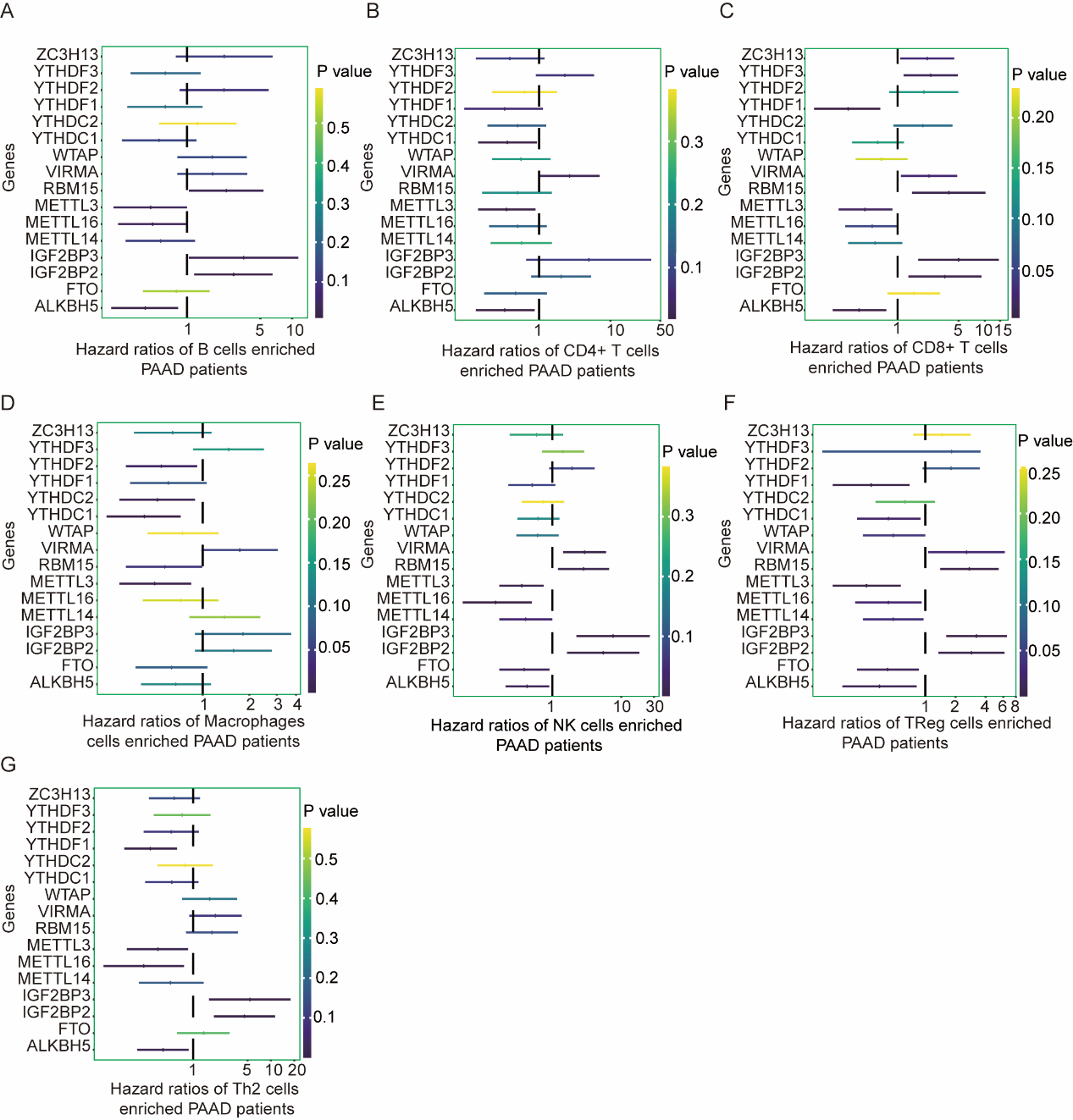


Figure S7. Forest plots of hazard ratios of survival associated with the high and low expression of m6A regulators in PAAD patients based on immune cells enriched subgroup. **(A-G)** Relationship between m6A regulators expression and overall survival of PAAD patients with B cells, CD4+ T cells, CD8+ T cells, macrophages cells, NK cells, Treg cells, and Th2 cells enriched.


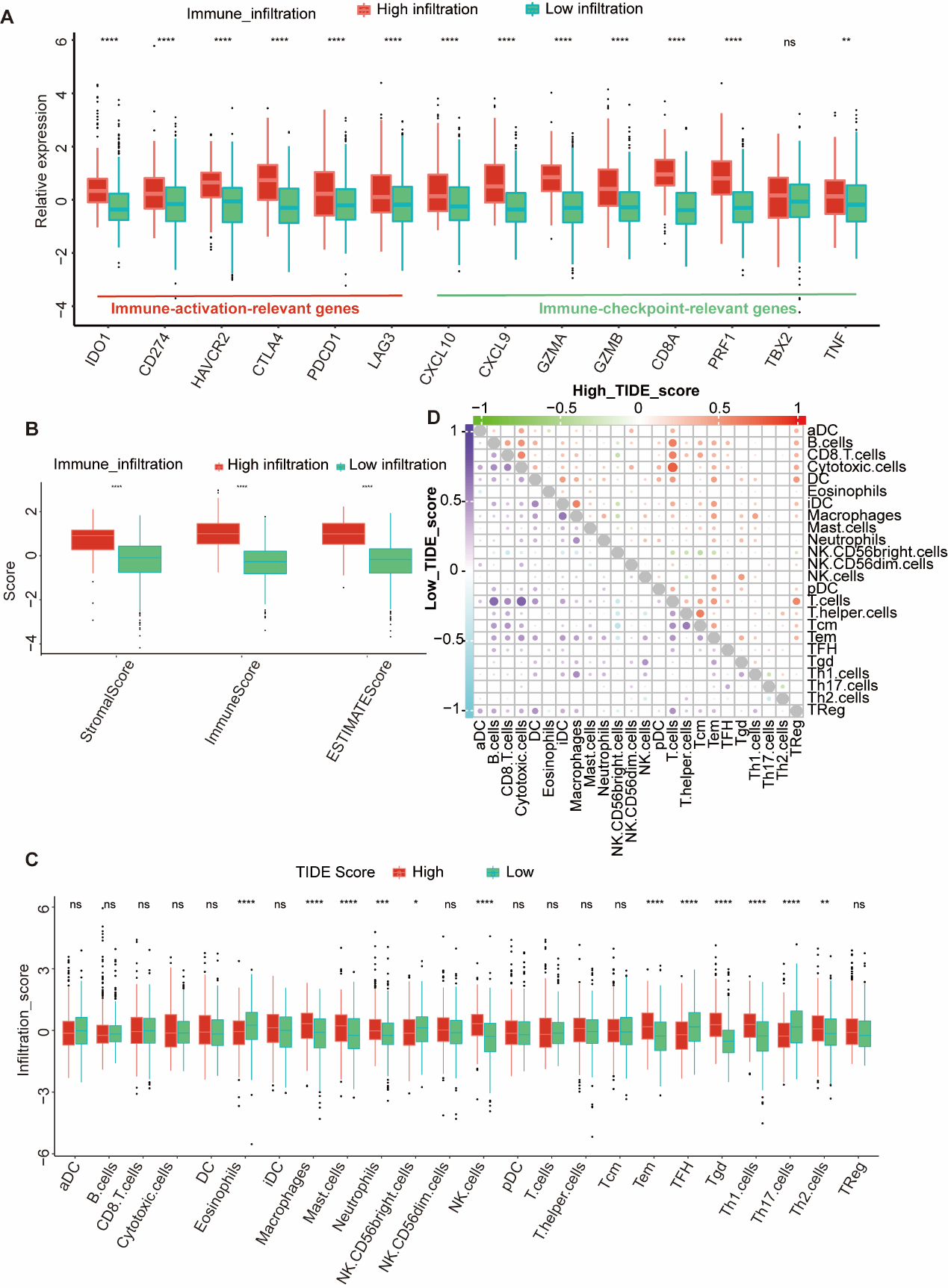


Figure S8. The correlation among immune infiltration, TIDE score, immune score, and immune-related genes in PAAD cohort. (A) Immune-activation-relevant genes and immune-checkpoint-relevant genes expressed in high and low immune infiltration subtypes. (B) ImmuneScore and StromalScore in high and low infiltration subtypes.

(C) The comparison of the fraction of tumor-infiltrating immune cells in high and low TIDE score groups. (D) The correlation of the infiltrating levels of tumor-infiltrating immune cells in high and low TIDE_score subtypes, respectively. Bubble size and color represents correlation coefficient r, don’t draw bubbles when p-value > 0.05. *p<0.05; **p<0.01; ***p<0.001; ****p<0.0001.


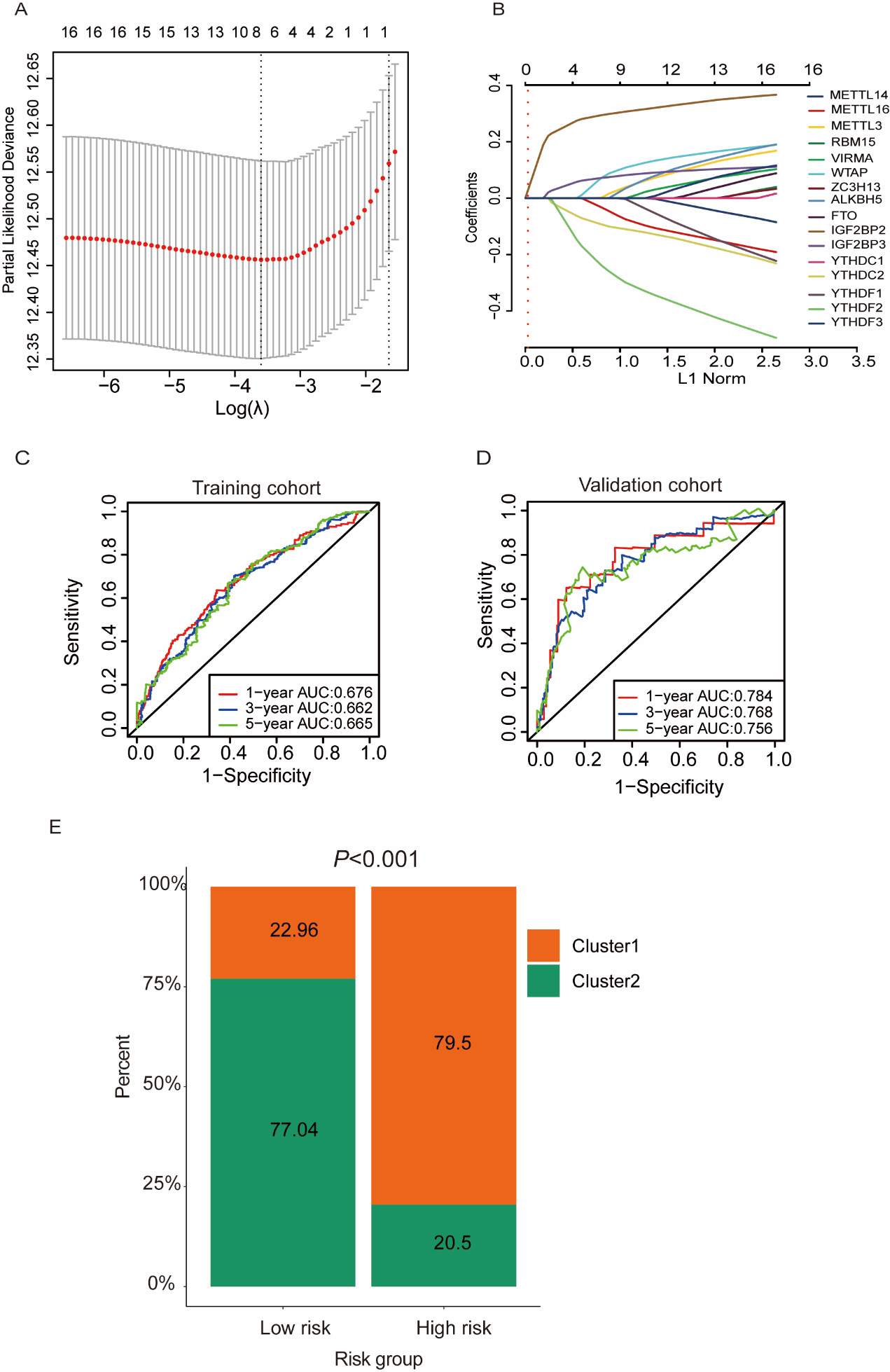


Figure S9. (A) Partial likelihood deviance for tuning the parameter selection in the LASSO regression model in PAAD training cohort. (B) LASSO coefficient profiles of the 16 m6A regulators in PAAD training cohort. (C-D) Receive operating characteristic curves of 1, 3, and 5 years based on the six m6A regulators signatures in PAAD training cohort (C) and validation cohort (D). (E) Cluster subtypes have significantly correlated with risk groups.


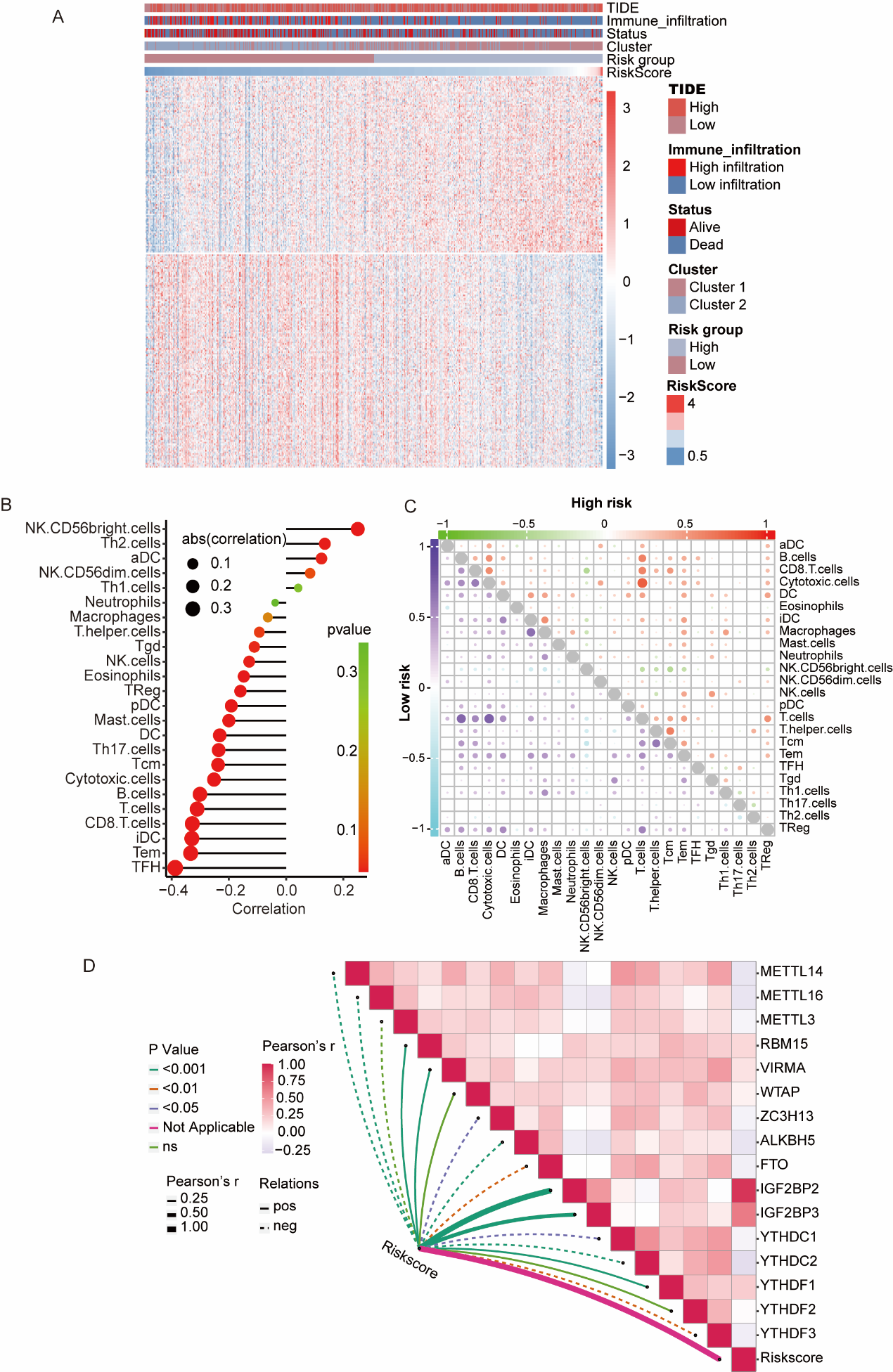


Figure S10. Correlation between riskscore and immune-related genes expression in PAAD. (A) The heatmap of correlation between riskscore and 378-immune-related genes. The resulted showed that 109 genes positively correlated and 132 genes negatively correlated with riskscore. The cutoff was set P<0.05. (B) Correlation between risk score and immune cells fraction in all PAAD samples. (C) Correlation between risk score and immune cells fraction in high and low risk subtypes PAAD patients, respectively. Bubble size and color represents correlation coefficient r, don’t draw bubbles when p-value > 0.05. (D) Correlation between risk score and expression of 16 m6A regulators.


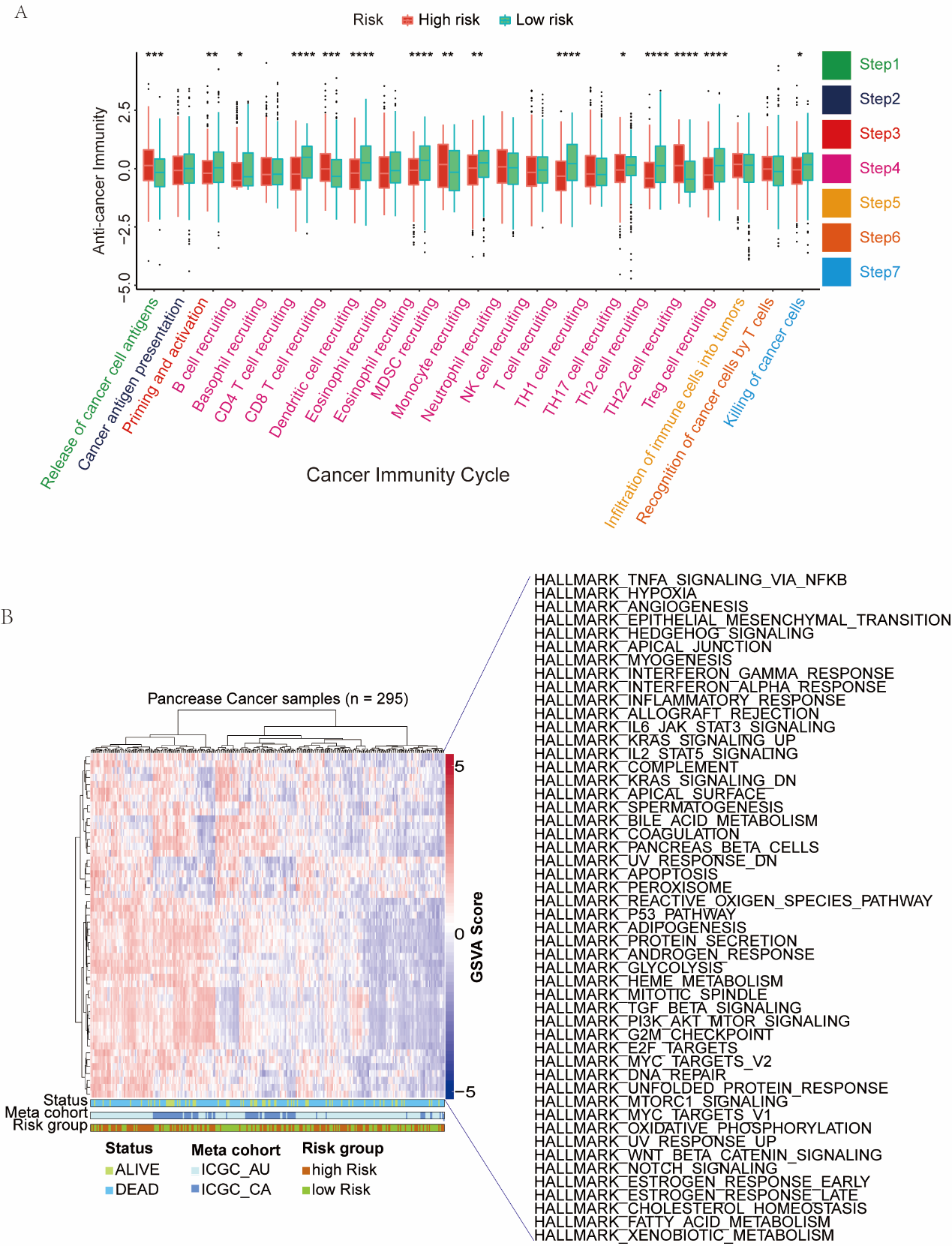


Figure S11. (A) Differences in the multiple steps of the cancer immunity cycle between high and low risk score in PAAD. (B) GSVA results of the validation cohort between high and low risk groups.


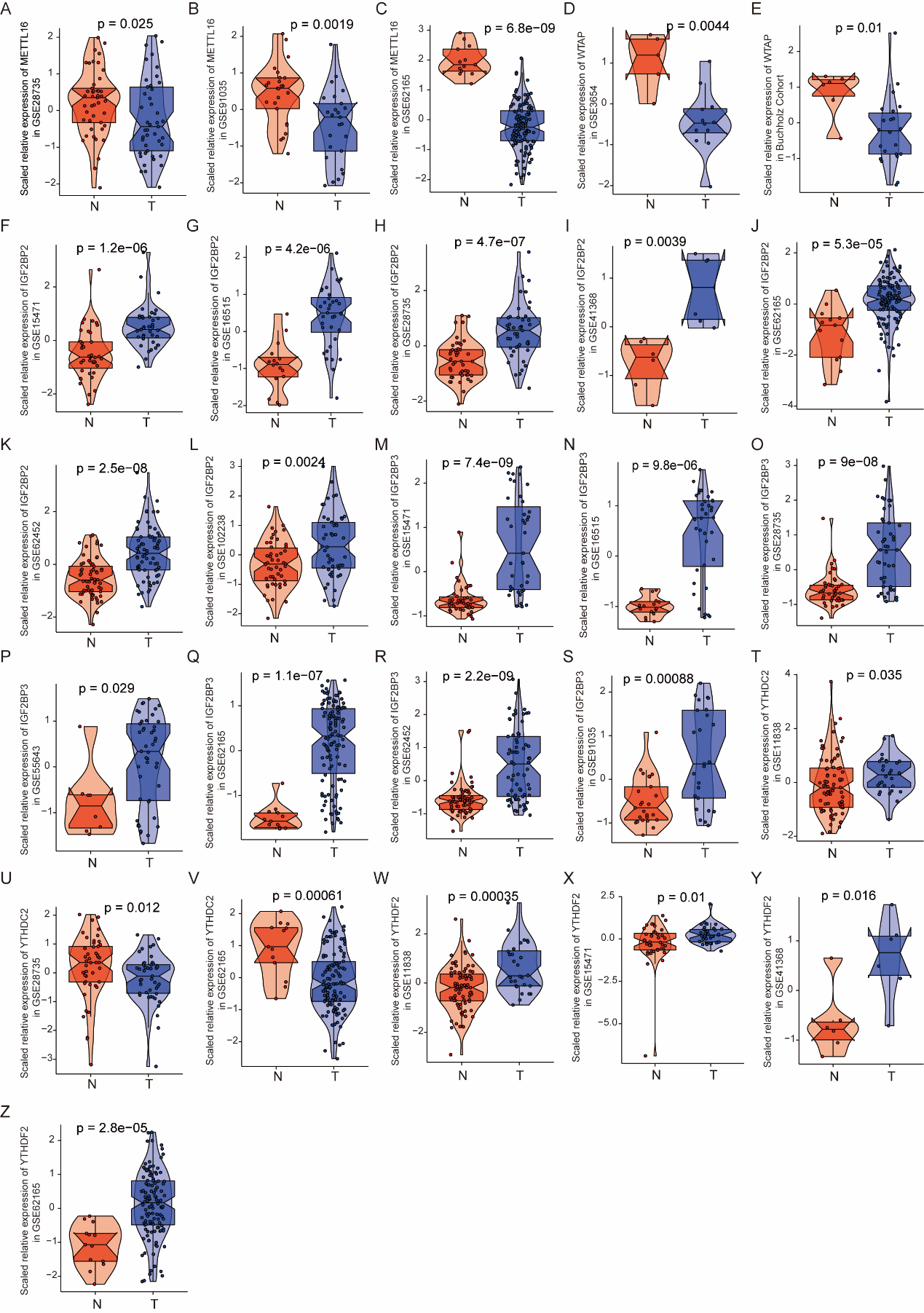


Figure S12. The violin plot showed the scaled relative expression levels of six-m6A-related genes in tumor (T) and normal (N) tissues of patients with pancreatic cancer in multiple datasets.
(A-C) METTL16; (D-E) WTAP; (F-L) IGF2BP2; (M-S) IGF2BP3; (T-V) YTHDC2; (W-Z) YTHDF2.


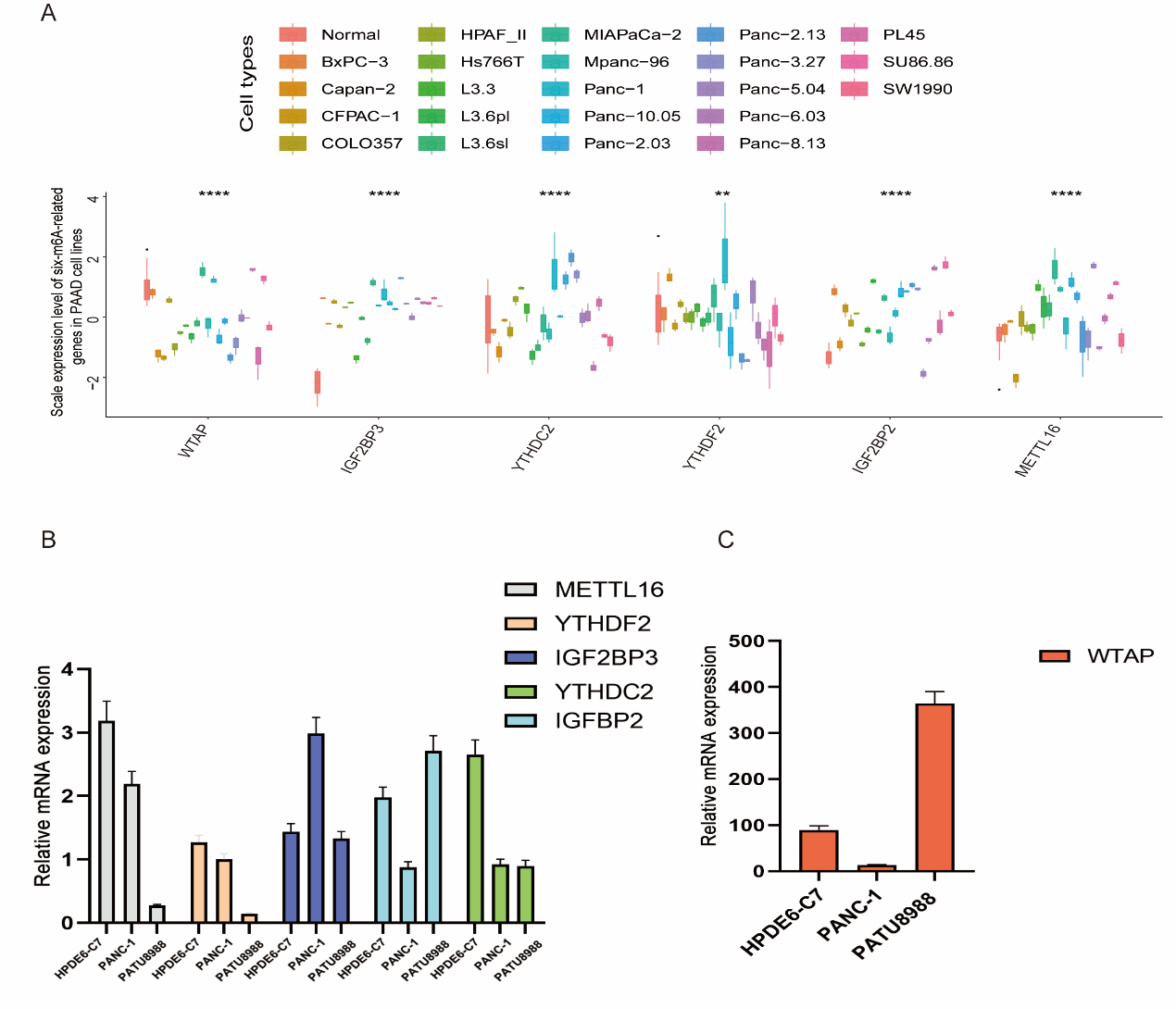


Figure S13. The scaled relative expression levels of six-m6A-related genes in pancreatic cancer cell lines and normal human pancreatic ductal epithelial cells.
(A) Scaled expression levels of six-m6A-related genes in multiple pancreatic cancer cell lines and normal human pancreatic ductal epithelial cells in GSE45757. (B) Relative expression level of METTL16, YTHDF2, IGF2BP2, IGF2BP3, YTHDC2, and IGF2BP2 in HPDE6-C7, PANC-1, and PATU8988 cell lines. (C) Relative expression level of WTAP in HPDE6-C7, PANC-1, and PATU8988 cell lines.
